# Avoidance response to CO_2_ in the lateral horn

**DOI:** 10.1371/journal.pbio.2006749

**Published:** 2019-01-17

**Authors:** Nélia Varela, Miguel Gaspar, Sophie Dias, Maria Luísa Vasconcelos

**Affiliations:** Champalimaud Research, Champalimaud Centre for the Unknown, Lisbon, Portugal; Stony Brook University, United States of America

## Abstract

In flies, the olfactory information is carried from the first relay in the brain, the antennal lobe, to the mushroom body (MB) and the lateral horn (LH). Olfactory associations are formed in the MB. The LH was ascribed a role in innate responses based on the stereotyped connectivity with the antennal lobe, stereotyped physiological responses to odors, and MB silencing experiments. Direct evidence for the functional role of the LH is still missing. Here, we investigate the behavioral role of the LH neurons (LHNs) directly, using the CO_2_ response as a paradigm. Our results show the involvement of the LH in innate responses. Specifically, we demonstrate that activity in two sets of neurons is required for the full behavioral response to CO_2_. Tests of the behavioral response to other odors indicate the neurons are selective to CO_2_ response. Using calcium imaging, we observe that the two sets of neurons respond to CO_2_ in a different manner. Using independent manipulation and recording of the two sets of neurons, we find that the one that projects to the superior intermediate protocerebrum (SIP) also outputs to the local neurons within the LH. The design of simultaneous output at the LH and the SIP, an output of the MB, allows for coordination between innate and learned responses.

## Author summary

Behavioral responses to a stimulus in the environment may be hardwired or acquired with experience. In the olfactory system of the fruit fly *Drosophila melanogaster*, associations of odors are formed in a brain structure called the mushroom body. A different brain region, the lateral horn, is thought to process innate responses, but this has not been shown directly. Here, we demonstrate that activity in neurons in the lateral horn does mediate innate responses. We used a behavioral experiment in which fruit flies avoid carbon dioxide to test the involvement in this response of different neurons in the lateral horn. We found that two sets of neurons, one local and one with projections to a separate region called the superior intermediate protocerebrum, mediate carbon dioxide response. These neurons do not mediate the avoidance response to the other aversive odors tested, indicating selectivity for carbon dioxide. By independent manipulation of and by recording from these two sets of neurons in the lateral horn, we revealed that the local neurons receive input from the output neurons. Thus, these output neurons of the lateral horn simultaneously output at the lateral horn and at the superior intermediate protocerebrum, which is also an area of mushroom body output. This connection between areas may allow coordination between innate and learned responses.

## Introduction

Animals use the olfactory system to find partners or food and to avoid predators. To a certain extent, the ability to navigate the olfactory environment is hardwired. This ability is expanded with life experiences that result in olfactory associations. The architecture of the olfactory system is comprehensively characterized in the fruit fly, and it is remarkably similar to the mammalian olfactory system [1,2]. Typically, each olfactory sensory neuron expresses a single odorant receptor that confers to the neuron its response profile [3–5]. With a few exceptions, the response profile of an odorant receptor is broad, meaning that an odor activates a combination of odorant receptors [6–10]. Olfactory sensory neurons expressing the same receptor project to the same glomerulus in the first olfactory center in the brain called the antennal lobe in the fly [3,4]. Most projection neurons (PNs) innervate a single glomerulus and carry the information to higher brain centers: the mushroom body (MB) and the lateral horn (LH) [11,12]. The MB is critical for olfactory associations [13]. The LH was ascribed the role of innate response based on MB silencing experiments [14,15]. PNs from the antennal lobe connect to the MB without apparent spatial selection, whereas at the LH, axonal arbors from different glomeruli interdigitate in a stereotyped fashion [11,12,16–19]. The stereotypy is consistent with the proposed role for the LH as the center for innate olfactory processing. While connectivity at the LH is being scrutinized, direct evidence for the functional role of the LH is still missing.

One of the strongest innate olfactory responses on a T-maze is the response to CO_2_. Unlike most insects, *Drosophila melanogaster* avoids CO_2_ when tested in a T-maze [20]. The aversive response may correspond to an avoidance of hypoxic environments, avoidance of exhalation of a large animal, or the recognition of the stress odor released by other flies. The aversive response up to 2% CO_2_ is solely mediated by antennal neurons, expressing the CO_2_ gustatory receptor (Gr) complex Gr21a-Gr63a, which connect to the V-glomerulus in the antennal lobe [7,21]. Synaptic inhibition of Gr21a-Gr63a–expressing neurons abolishes the avoidance response to low concentrations of CO_2_ [20]. Conversely, artificial stimulation of CO_2_-sensing neurons with light elicits the avoidance behavior typically observed in response to CO_2_ [22]. Three PNs that innervate the V-glomerulus (VPNs) clearly project to the LH [17,18,23]. One VPN innervates only the LH, the second one innervates the LH and the MB calyx on both brain hemispheres (biVPN or PNv-1), and the third one connects to the LH and the superior intermediate protocerebrum (SIP) (PNv-3). PNv-3 dendrites innervate many glomeruli in the antennal lobe. It was shown that this PN gates the behavioral response of the biVPN, allowing a behavioral response at 0.5% CO_2_ but not at 2% CO_2_. A number of other PNs connect the V-glomerulus with different brain areas. Notably, PNv-2 has dendrites in the V-glomerulus alone and connects to the SIP, an area also innervated by the MB output neurons [24,25]. PNv-2 participates in the avoidance response to 2% CO_2_. The complexity of connectivity at higher brain levels is probably related with the double valence of CO_2_. CO_2_ generates an aversive response in a T-maze, but it is a product of fermentation, therefore indicating the presence of food. Starvation changes how the CO_2_ response is processed. Under starvation, the MB activity is required for the response, but not in fed flies [26].

Not all responses to CO_2_ are mediated by Gr21a-Gr63a neurons. Higher CO_2_ concentrations elicit an aversive response to acid that is processed in a separate glomerulus [27]. Also, a recent study shows that the response to CO_2_ is state dependent, with high-activity flies moving towards CO_2_ and low-activity flies avoiding it [28]. These results explain previous reports that CO_2_ can elicit an attractive response in flying individuals [29,30]. The attractive response does not require Gr21a-Gr63a receptors; instead, it is mediated by the ionotropic receptor (IR) 25a[28].

Here, we address directly the behavioral role of the LH neurons (LHNs) using the CO_2_ avoidance on a T-maze to low concentrations to probe the requirement of the LH for innate responses. We demonstrate that activity in two sets of neurons is required selectively in the behavioral response to CO_2_. Using calcium imaging, we observe the neurons responding to CO_2_ in a different concentration-dependent manner. Activity manipulation experiments combined with calcium imaging reveal that the two sets of neurons are connected in a circuit that receives input from the V-glomerulus.

## Results

### Neurons labeled by lines *21G11* and *23C09* process CO_2_ avoidance

**Fig 1:**
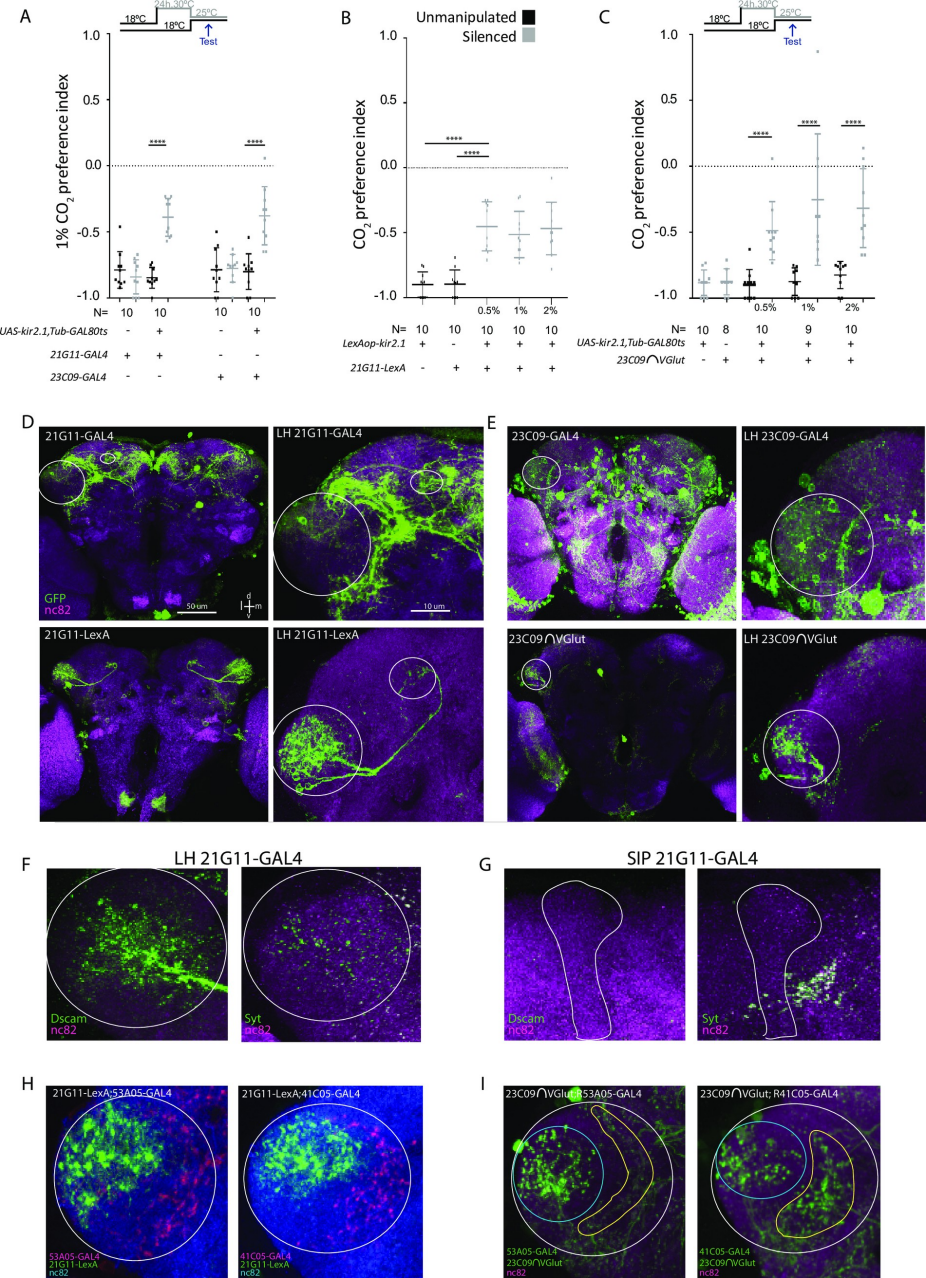
Activity in *21G11* and *23C09* neurons is required for behavioral response to CO_2_. (A) T-maze response to 1% CO_2_ of *21G11-GAL4* and *23C09-GAL4* flies driving *UAS-Kir2*.*1*, *TubGAL80*^*TS*^ expression. (A and C) Black dots, no heat induction of Kir2.1 expression (see Materials and Methods). Gray dots, heat induction of Kir2.1 expression before test. The top and bottom lines represent the first and the third quartiles. The line across the box is the median. (B and C) T-maze response to three CO_2_ concentrations—0.5%, 1%, and 2%—of the flies with *21G11-LexA* driving *LexAopKir2*.*1* expression (B) and *23C09∩VGlut* driving *UAS-Kir2*.*1*,*TubGAL80*^*TS*^ expression (C). For *21G11-LexA* driving *LexAopkir2*.*1* expression, black dots represent parental controls, and gray dots represent constitutive Kir2.1 expression. For *23C09∩VGlut* driving *UAS-Kir2*.*1*, *TubGAL80*^*TS*^ expression, black dots represent no heat induction of Kir2.1 expression, and gray dots represent both parental controls and heat induction of Kir2.1 expression before test. Post hoc two-way ANOVA showed no significance when comparing among expressions for both control and test samples. (D) Brain and LH *UAS-mCD8-GFP* expression of *21G11-GAL4* and *21G11-LexA*. (E) Brain and LH *UAS-mCD8-GFP* expression of *23C09-GAL4* and *23C09∩VGlut*. (D–E) Circle highlights the LH, and oval highlights the SIP. (F and G) Dscam17.1-GFP and syt-HA expression in the LH and SIP of *21G11-GAL4* (green). In (F), circle highlights the LH. In (G), the vertical lobe of the MB is drawn to facilitate visualization of the adjacent SIP. (H) LH showing expression of *21G11-LexA* (green) and the VPN lines *53A05-GAL4* (red) and *41C05-GAL4* (red). Circle highlights the LH. (I) LH expression of *23C09∩VGlut* (blue circles) and the VPN lines *53A05-GAL4* (yellow line) and *41C05-GAL4* (yellow line). White circle highlights the LH. For all images, the brain neuropil was stained with nc82 (magenta and blue). ±SEM ****p < 0.0001. All *p* values are calculated with one-way ANOVA. d, dorsal; Dscam17.1, Down syndrome cell adhesion molecule with isoform 1 of the transmembrane domain; l, lateral; LH, lateral horn; m, medial; MB, mushroom body; nc82, monoclonal antibody to Bruchpilot; PN, projection neuron; SEM, standard error of the mean; SIP, superior intermediate protocerebrum; v, ventral; VPN, V-glomerulus–innervating PN.

We chose to investigate the role of LHNs in the context of the response to CO_2_ because of the strength of the innate response on a T-maze. To identify neurons involved in CO_2_ avoidance, we performed an inhibitory screen of fly lines labeling LHNs (S1 Fig). Through visual inspection of the expression pattern of Janelia’s collection of *GAL4* lines, we selected 32 lines with obvious LH innervation [31,32]. To silence the neurons, we expressed the inward-rectifier potassium channel Kir2.1 [33], which hyperpolarizes neurons and thus decreases the probability of firing an action potential. In the screen and in other behavioral experiments with GAL4 lines, we restricted Kir2.1 expression to the adult stage by using a temperature-sensitive GAL80 (GAL80^TS^, see Materials and Methods) [34]. The 32 lines were tested on a T-maze in which flies were allowed to choose between air and 0.5% CO_2_ (S1A Fig). Eight lines showed a significant reduction in avoidance (multiple *t* test corrected with Holm-Sidak method, *p* < 0.05,) and when retested, three of them exhibited a consistent reduction in avoidance to CO_2_ (S1B Fig). Line *65D12* was discarded because of innervation in the V-glomerulus (see laser scanning microscope file at http://flweb.janelia.org/cgi-bin/view_flew_imagery.cgi?line=R65D12). Neurons in lines *21G11* and *23CO9* (which we will henceforth call *21G11* and *23CO9* neurons) are necessary for the behavioral response to CO_2_ (Fig 1A, here tested to 1% CO_2_). We observe a similar reduction in the behavioral response to CO_2_ when we express Kir2.1 only in the brain neurons labeled by these lines (S2A and S2B Fig). Since the requirement of the biVPNs and the MB for CO_2_ avoidance is feeding-state dependent [18], we tested whether feeding state also affects the contribution of *21G11* and *23C09* neurons in the avoidance response of the fly. We observe that starvation does not alter the phenotype, indicating that the involvement of *21G11* and *23C09* neurons in CO_2_ response is independent of the feeding state of the fly (S2C Fig).

Given that CO_2_ avoidance is reduced but not abolished for either line, we tested flies with both sets of neurons silenced (S2D Fig). We observe no change in the phenotype, indicating that the two sets of neurons do not complement each other, i.e., the activity of these populations may not be independent to drive avoidance responses (post hoc two-way ANOVA comparing individual and combined expressions not significant both for control and test samples). It has been previously shown that different PNs of the V-glomerulus are required for the behavioral response to different CO_2_ concentrations [17]. Therefore, we tested whether the requirement of *21G11* and *23C09* neurons for avoidance to CO_2_ was concentration dependent. For this experiment, we used lines *21G11-LexA* and *23C09∩VGlut*, which have a restricted expression when compared to the *21G11-GAL4* and *23C09-GAL4* lines, respectively (Fig 1B and 1C, see below). We observe that silencing the activity of these neurons reduces the avoidance behavior of the flies in a comparable manner across odor concentrations (post hoc two-way ANOVA comparing across odor concentrations not significant, both for control and test samples). These results suggest that *21G11* and *23C09* neurons contribute to CO_2_ avoidance independently of concentration (within the range that does not engage the acid-sensing response).

Anatomical inspection reveals that line *21G11* labels one cluster of neurons that innervate the dorsoposterior area of the LH and project to the SIP (Fig 1D). The *21G11* cluster, with its posterior cell bodies and projections to the SIP, appears to correspond to the posterior ventral (PV) 5a tract described by Frechter and colleagues [35]. We generated a *LexA* version of the line to allow independent manipulation of the *21G11-LexA* neurons and other neurons labeled with *GAL4*. The *LexA* version of *21G11* is very sparse. Additionally, the number of neurons labeled in the LH cluster is smaller. We counted 10 cell bodies in the *LexA* version and 16 to 18 cell bodies in the *GAL4* version (*n* = 5). When we overlaid the expression of both lines, we found that seven to nine cells were specific to *21G11-GAL4*, three cells are specific to *21G11-LexA*, and seven cells are common to both lines (S3 Fig, *n* = 9). Nevertheless, activity in *21G11-LexA* neurons is necessary to elicit full CO_2_ avoidance (Fig 1B). Line *23C09* labels more than one cluster of neurons at the LH (Fig 1E). To narrow down the expression of line *23C09*, we generated a *splitGAL4* version, and then we intersected the expression with that of different neurotransmitter–*splitGAL4* lines [36,37]. We found that a glutamatergic cluster located posteriorly is involved in the response (Fig 1C–1E). This cluster, which we will call *23C09∩VGlut*, has 8 to 10 cell bodies (*n* = 3) with processes only within the LH. It appears to correspond to the primary neurite tract PV4a described in Frechter and colleagues [35]. For expression of restricted lines in 10 μm sections across the brain and ventral nerve cord (VNC) expression in all lines, see S4 Fig. In order to determine the polarity of *21G11* and *23C09∩VGlut* neurons, we used the neural compartment markers Down syndrome cell adhesion molecule with isoform 1 of the transmembrane domain (Dscam17.1)-green fluorescent protein (GFP) [38] for dendrites and synaptotagmin-hemagglutinin (HA) [39] for presynaptic areas. Dscam17.1-GFP signal localized exclusively to the LH in *21G11*, which indicates that these neurons receive inputs there, presumably olfactory. The synaptotagmin-HA signal, on the other hand, is localized both to the LH and the SIP. To exclude the possibility that the *GAL4* cluster holds a mixed population of neurons with different polarities, we marked the more restricted *21G11-LexA* neurons and observed the same distribution of synaptotagmin-HA (S5A Fig). These results suggest that *21G11* neurons output both in the SIP and the LH. For *23C09∩VGlut* neurons, the signal is localized in the LH for both Dscam17.1-GFP and synaptotagmin-HA (S5B Fig). Finally, we asked whether *21G11* or *23C09* contact projections from the V-glomerulus. Three distinct VPNs at the antennal lobe innervate the medial border of the LH [17,18]. We tested two VPNs for which there are lines available with a strong visible projection. We do not see clear overlap at the LH between the innervation of the VPNs and the innervation of the LHNs we identified (Fig 1H and 1I). This observation, together with the fact that the reduction in avoidance is not complete, indicates that additional LHNs are involved in the response.

### *21G11* and *23C09∩VGlut* neurons respond to CO_2_ in different concentration-dependent manners

**Fig 2:**
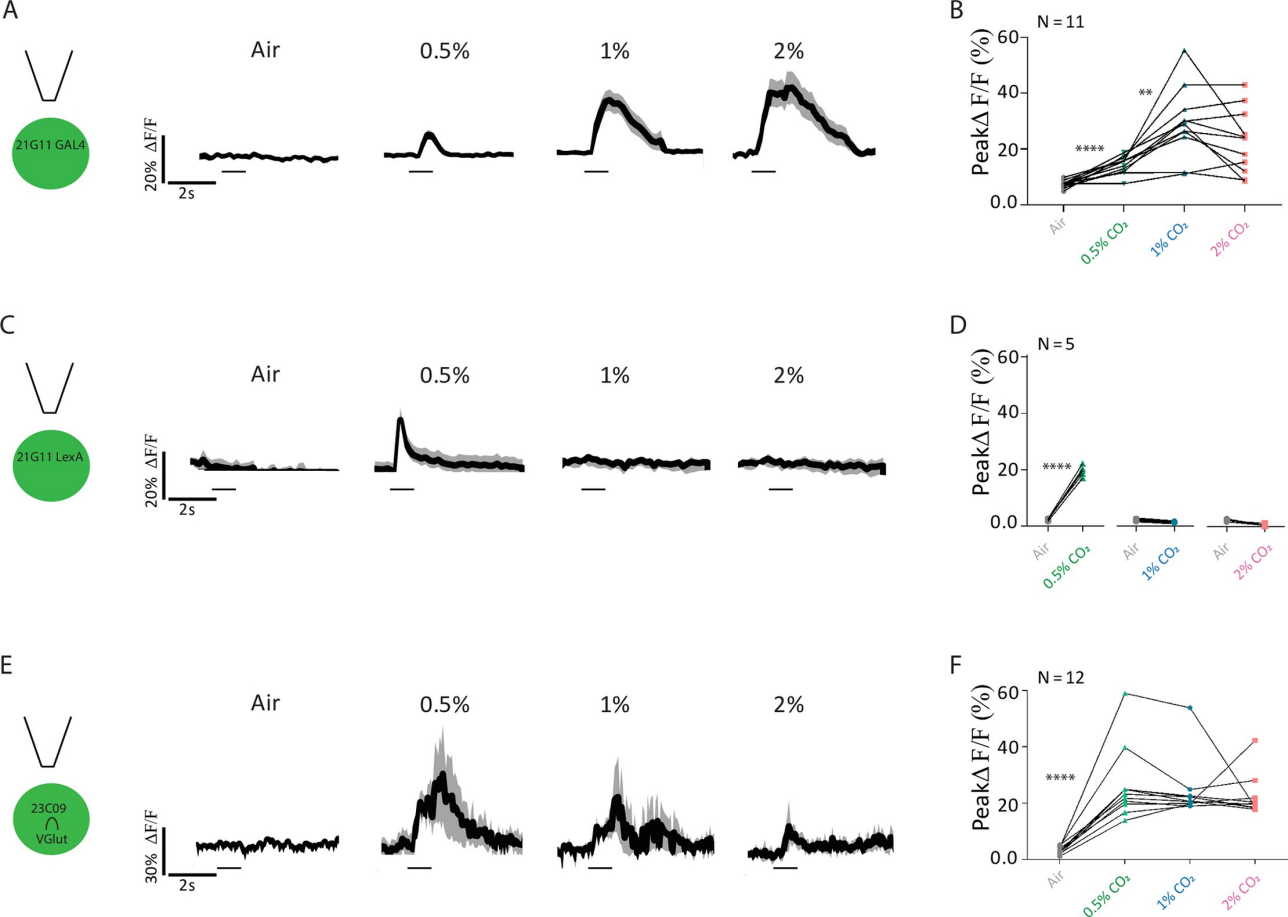
Physiological response to CO_2_ of *21G11* and *23C09* neurons. (A, C, and E) LH activity of *21G11-GAL4*, *21G11-LexA* and *23C09∩VGlut*, respectively, to air, 0.5%, 1%, and 2% of CO_2_. Schematics of the experiment and average time course of GCaMP6m intensity change. The black bar indicates the time of the stimulus. The black trace represents the average, while the gray shows the range of individual traces. (B, D, and F) Peak GCaMP6m intensity change after stimulation with air, 0.5%, 1%, and 2% of CO_2_. **p < 0.01, ****p < 0.0001. Comparisons are made between tested concentrations. All *p* values are calculated with Wilcoxon signed-rank test. LH, lateral horn.

Having demonstrated that *21G11* and *23C09* neurons are required for the behavioral response to CO_2_, we next addressed the physiological response of these neurons. We measured the changes in internal free-calcium levels in *21G11* and *23C09∩VGlut* neurons upon stimulation with CO_2_. For this, we expressed the genetically encoded calcium indicator GCaMP6m [40] in *21G11* and *23C09∩VGlut* neurons and recorded the calcium dynamics in a live fly preparation at the two-photon microscope. Within each LH imaged, we manually delineated the region of interest as the area around the observable innervation to measure the changes in fluorescence. The neurons labeled by *21G11* respond to all concentrations of CO_2_, with the peak ΔF/F increasing from 0.5 to 1% CO_2_ (Fig 2A and 2B, Wilcoxon signed-rank test w = 66.00, *p* = 0.0011). The peak response to 1% and 2% are not significantly different, though the length of the response appears to be larger at 2% (Wilcoxon signed-rank test w = 38.00, *p* = 0.0727). We also tested responses to CO_2_ in the subset of *21G11* neurons labeled by *21G11-LexA* (Fig 2C and 2D). To our surprise, this subset responds only to 0.5% CO_2_ (Wilcoxon signed-rank test w = 15.00, *p* < 0.0001). This observation suggests that within the *21G11* cluster, different neurons are sensitive to different concentrations of CO_2_. However, in Fig 1C, we observed that when these same neurons are silenced, the behavioral response to different CO_2_ concentrations does not change. We will address this discrepancy between physiology and behavioral requirement three sections below. Recordings of *23C09∩VGlut* neurons show that they respond to all CO_2_ concentrations tested. Though the curve of the response appears larger for lower concentrations, there is no significant difference between peak amplitudes of ΔF/F of different concentrations (Fig 2E and 2F, 0.5%–1%: Wilcoxon signed-rank test w = 71.00, *p* = 0.1801; 1%–2%: Wilcoxon signed-rank test w = 68.00, *p* = 0.6377). In summary, *21G11-GAL4* and *23C09∩VGlut* clusters respond to CO_2_ stimulation at different concentrations, with each set of neurons exhibiting a different pattern of free calcium response to CO_2_ stimulation.

### Physiological response of *23C09∩VGlut* depends on output of *21G11-LexA*

**Fig 3:**
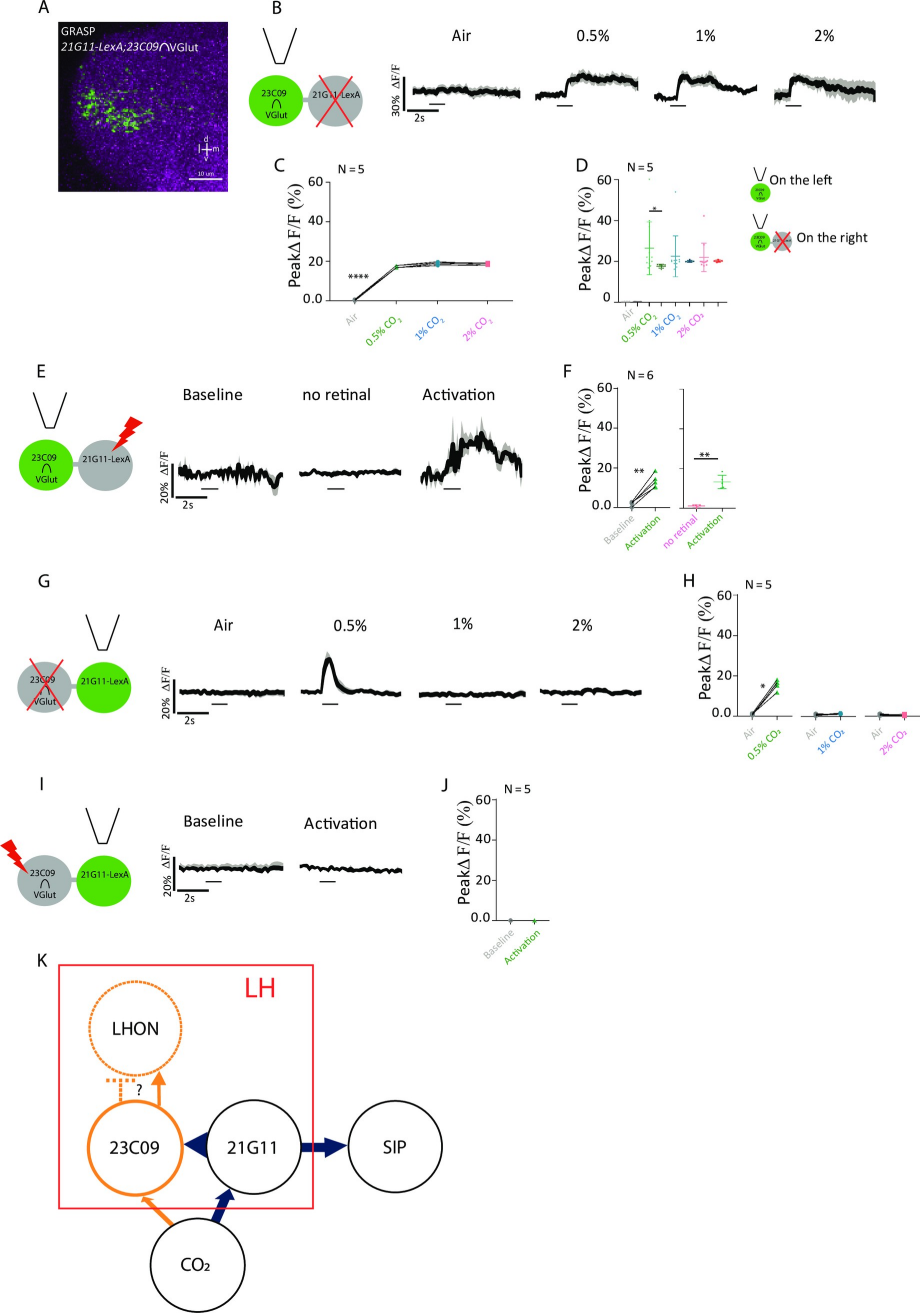
*21G11-LexA* neurons are presynaptic to *23C09*∩*VGlut* neurons. (A) LH showing GRASP, a measure of contact between the membranes of neurons, in *21G11-LexA* and *23C09∩VGlut*. (B) Schematics of the experiment and calcium response at the LH, using GCaMP6m, of *23C09∩VGlut* neurons to air, 0.5%, 1%, and 2% of CO_2_ while *21G11-LexA* neurons are silenced by expression of Kir2.1. (C) Peak GCaMP6m intensity change after stimulation with air, 0.5%, 1%, and 2% of CO_2_. (D) Peak GCaMP6m intensity change between Fig 2F and Fig 3C after stimulation with air, 0.5%, 1%, and 2% of CO_2_. (E) Schematics of the experiment and calcium response at the LH of *23C09∩VGlut* during baseline, activity without retinal, and upon activation with 720 nm light of *21G11-LexA* driving expression of Chrimson. (F) Peak GCaMP6m intensity change of *23C09∩VGlut* during baseline, activity without retinal, and upon activation with 720 nm light of *21G11-LexA* driving expression of Chrimson. (G) Schematics of the experiment and calcium response at the LH, using GCaMP6m, of *21G11-LexA* neurons to air, 0.5%, 1%, and 2% of CO_2_ while *23C09∩VGlut* neurons are silenced by expression of Kir2.1. (H) Peak GCaMP6m intensity change after stimulation with air, 0.5%, 1%, and 2% of CO_2_. (I) Schematics of the experiment and LH activity of *21G11-LexA* upon activation of *23C09∩VGlut* neurons, expressing Chrimson, with 720 nm light. (J) Peak GCaMP6m intensity change of *21G11-LexA* during baseline and activation with 720 nm light of *23C09∩VGlut* driving expression of Chrimson. (K) Proposed model of the LHNs processing CO_2_ information. Elements in dashed lines are hypothetical. For (B), (E), (G), and (I), the average time course of GCaMP6m intensity change is shown. The black bar indicates the time of the stimulus. Scale bar = 10 μm. *p < 0.05, **p < 0.01, ****p < 0.0001. All *p* values are calculated with Wilcoxon signed-rank test. d, dorsal; GFP, green fluorescent protein; GRASP, GFP reconstitution across synaptic partners; l, lateral; LH, lateral horn; LHN, LH neuron; LHON, LH output neuron; m, medial; SIP, superior intermediate protocerebrum; v, ventral.

The two sets of neurons that we identified innervate a similar region of the LH and contribute to the same behavioral response. When we silenced both clusters simultaneously, we saw no additive effect, indicating they are not independent from each other to drive avoidance (S2D Fig, post hoc two-way ANOVA comparing individual and combined expressions not significant both for control and test samples). We therefore asked whether the two clusters are connected. To this end, we used GFP reconstitution across synaptic partners (GRASP), which reveals membrane contact between two sets of neurons [41]. We observe a strong signal in the LH, indicating that the membranes of the two clusters contact each other (Fig 3A). To assess functional connectivity, we manipulated the activity of one cluster while imaging activity on the second cluster. We silenced *21G11-LexA* neurons with the expression of Kir2.1 using the *LexA/LexAOp* expression system and imaged *23C09∩VGlut* neurons expressing GCaMP6m with the *GAL4/UAS* system (schematic in Fig 3B). To control for silencing, we coexpressed Kir2.1 and GCaMP6m in *21G11-LexA* neurons and confirmed that no calcium signal is observed with CO_2_ stimulation (S6A and S6B Fig). Upon presentation of CO_2_ at the concentrations 0.5%, 1%, and 2%, we observed a very consistent response across trials and across concentrations in *23C09∩VGlut* neurons when *21G11-LexA* neurons are silenced (Fig 3B and 3C, Mann–Whitney, not significant). When we compared the peak responses of *23C09∩VGlut* while *21G11-LexA* is intact (Fig 2F) or silenced (Fig 3C), we found that there is pronounced reduction for 0.5% CO_2_ responses, which corresponds to the profile of *21G11-LexA* responses (Fig 3D, Wilcoxon signed-rank test w = 17.00, *p* = 0.0337). The results indicate that the output of *21G11-LexA* neurons contributes to *23C09∩VGlut* activity. To test this directly, we expressed the red-shifted channelrhodopsin Chrimson [42] in *21G11-LexA* neurons to allow activation of *21G11-LexA* neurons with 720 nm light while recording *23C09∩VGlut* calcium concentration with GCaMP6m. We observe that indeed, activation of *21G11-LexA* neurons with light generates a strong calcium response in *23C09∩VGlut* neurons (Fig 3E and 3F, Mann–Whitney, U = 94, *p* = 0.0012). No calcium response was observed in *23C09∩VGlut* neurons when flies were not fed retinal, which is necessary for Chrimson function (Fig 3E and 3F, Mann–Whitney, not significant). To check that Chrimson indeed activates *21G11-LexA* neurons, we expressed both Chrimson and GCaMP6m in these neurons and could see a response with light stimulation (S6C and S6D Fig, Mann–Whitney, U = 63, *p* = 0.0321).

We next did the converse experiments in which we manipulate activity in *23C09∩VGlut* neurons and image the activity in *21G11-LexA* neurons, using the same tools with the expression systems reversed. Silencing *23C09∩VGlut* neurons does not change *21G11-LexA* response to CO_2_ presentation (Fig 3G and 3H, Air–0.5%: Wilcoxon signed-rank test w = 15.00, *p* = 0.0002). These results indicate that *23C09∩VGlut* neurons do not output into *21G11-LexA* neurons. To control for silencing, we coexpressed Kir2.1 and GCaMP6m in *23C09∩VGlut* neurons and confirmed that no calcium signal is observed with CO_2_ stimulation (S7A and S7B Fig, Wilcoxon signed-rank test not significant). We then performed optogenetic activation of *23C09∩VGlut* neurons and recorded the calcium response of *21G11-LexA* neurons. We did not observe a calcium response in *21G11-LexA* neurons upon light stimulation of *23C09∩VGlut* neurons (Fig 3I and 3J, Mann–Whitney not significant). To control for activation of *23C09∩VGlut* neurons, we expressed both Chrimson and GCaMP6m in these neurons and could see a response with light stimulation (S7C and S7D Fig, Mann–Whitney, U = 67, *p* = 0.0361). The activation results further support the notion that *23C09∩VGlut* does not output into *21G11-LexA* neurons.

Taken together, these results indicate that *21G11-LexA* neurons are presynaptic to *23C09∩VGlut* neurons. The presence of a presynaptic marker at the LH processes of *21G11-LexA* neurons is consistent with these observations (Fig 1F). Based on our findings, we propose a model in which there are two outputs to the CO_2_ response (Fig 3K). *21G11-LexA* neurons outputs at the LH to activate *23C09∩VGlut* local neurons. These, in turn, activate or inhibit another set of LH output neurons that will initiate a motor response. *21G11-LexA* neurons also output at the SIP, where coordination with the MB signal is likely taking place.

### *21G11-LexA* neurons receive inputs from the V-glomerulus

**Fig 4:**
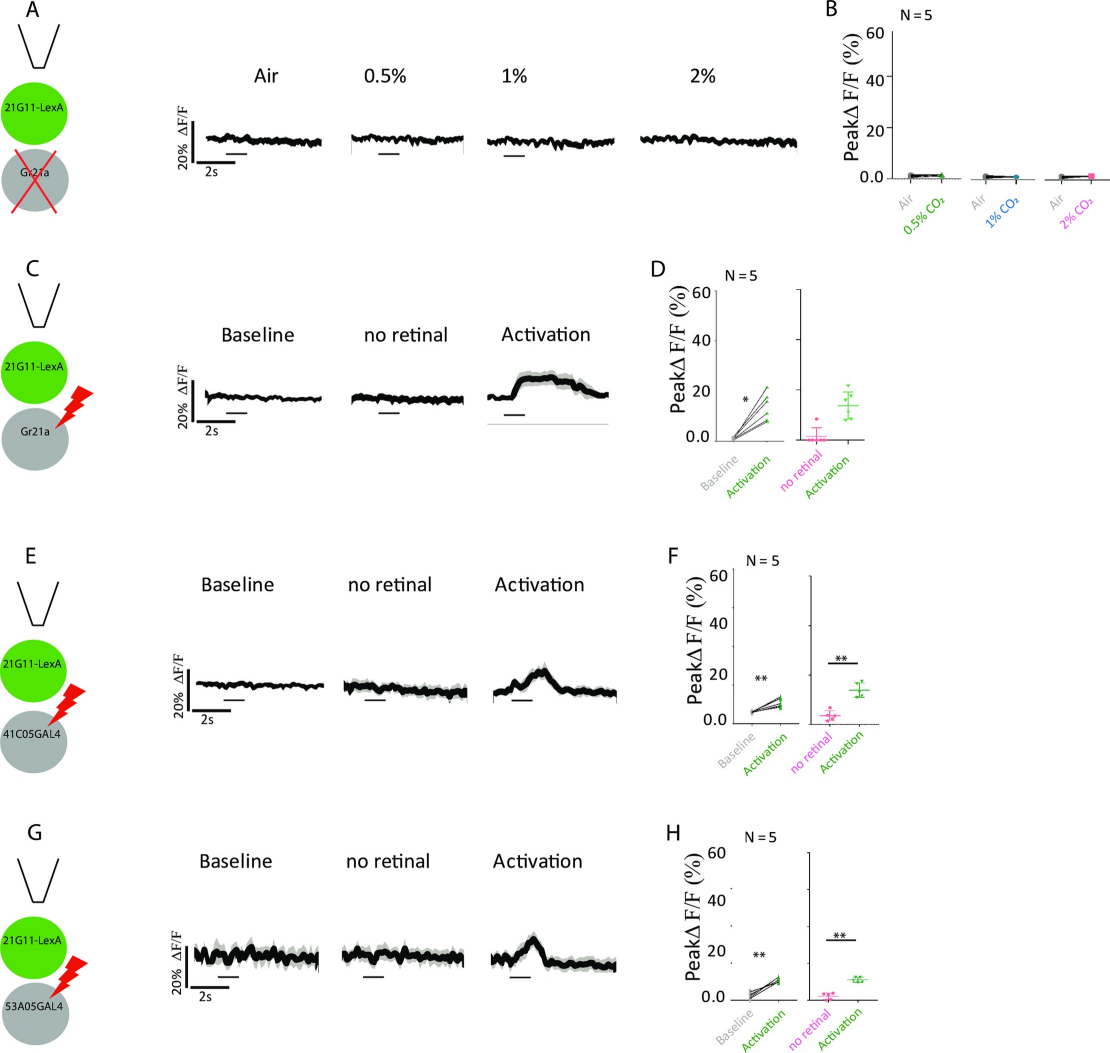
*21G11-LexA* neurons receive input from the V-glomerulus. (A) Schematics of the experiment and calcium response at the LH, using GCaMP6m, of *21G11-LexA* neurons to air, 0.5%, 1%, and 2% of CO_2_ while *Gr21a-GAL4* neurons are silenced by expression of Kir2.1. (B) Peak GCaMP6m intensity change after stimulation with air, 0.5%, 1%, and 2% of CO_2_. (C, E, and G) Schematics of the experiment and calcium response at the LH of *21G11-LexA* neurons during baseline, activity without retinal, and upon activation with 720 nm light of *Gr21a-GAL4* (C), *41C05-GAL4* (E), and *53A05-GAL4* (G) driving expression of Chrimson. (D, F, and H) Peak GCaMP6m intensity change of *21G11-LexA* during baseline, activity without retinal, and upon activation with 720 nm light of *Gr21a-GAL4* (D), *41C05-GAL4* (F), and *53A05-GAL4* (H) driving expression of Chrimson. For (A), (C), (E), and (G), the average time course of GCaMP6m intensity change is shown. The black bar indicates the time of the stimulus. Gr, gustatory receptor; LH, lateral horn.

We wished to confirm that the CO_2_ responses we observe originate in the V-glomerulus because the CO_2_ response is flexible and may have different origins[28]. We silenced the neurons expressing the CO_2_ coreceptor Gr21a that converge in the V-glomerulus while we recorded the response to CO_2_ in *21G11-LexA* neurons using similar methodology to that used in the previous section. We find that CO_2_ response is abolished in *21G11-LexA* neurons (Fig 4A and 4B, Wilcoxon signed-rank test not significant). Conversely, optogenetic activation of Gr21a neurons induces activation of *21G11-LexA* neurons (Fig 4C and 4D, Mann–Whitney, U = 86, *p* = 0.0172). We found that VPNs that innervate specifically the V-glomerulus and project to the LH do not directly synapse onto *21G11-LexA* (Fig 1H). We asked whether, even so, they induce activation of *21G11-LexA*. Stimulation of each VPN leads to activation of *21G11-LexA* neurons (Fig 4F and 4H, 4F: Mann–Whitney, U = 78, *p* = 0.0188; 4H: Mann–Whitney, U = 82, *p* = 0.0124). Our results indicate that *21G11-LexA* neurons are an integral part of a CO_2_ response that originates in the V-glomerulus.

### Output of *21G11-LexA* neurons regulates activity of other *21G11-GAL4* neurons

**Fig 5:**
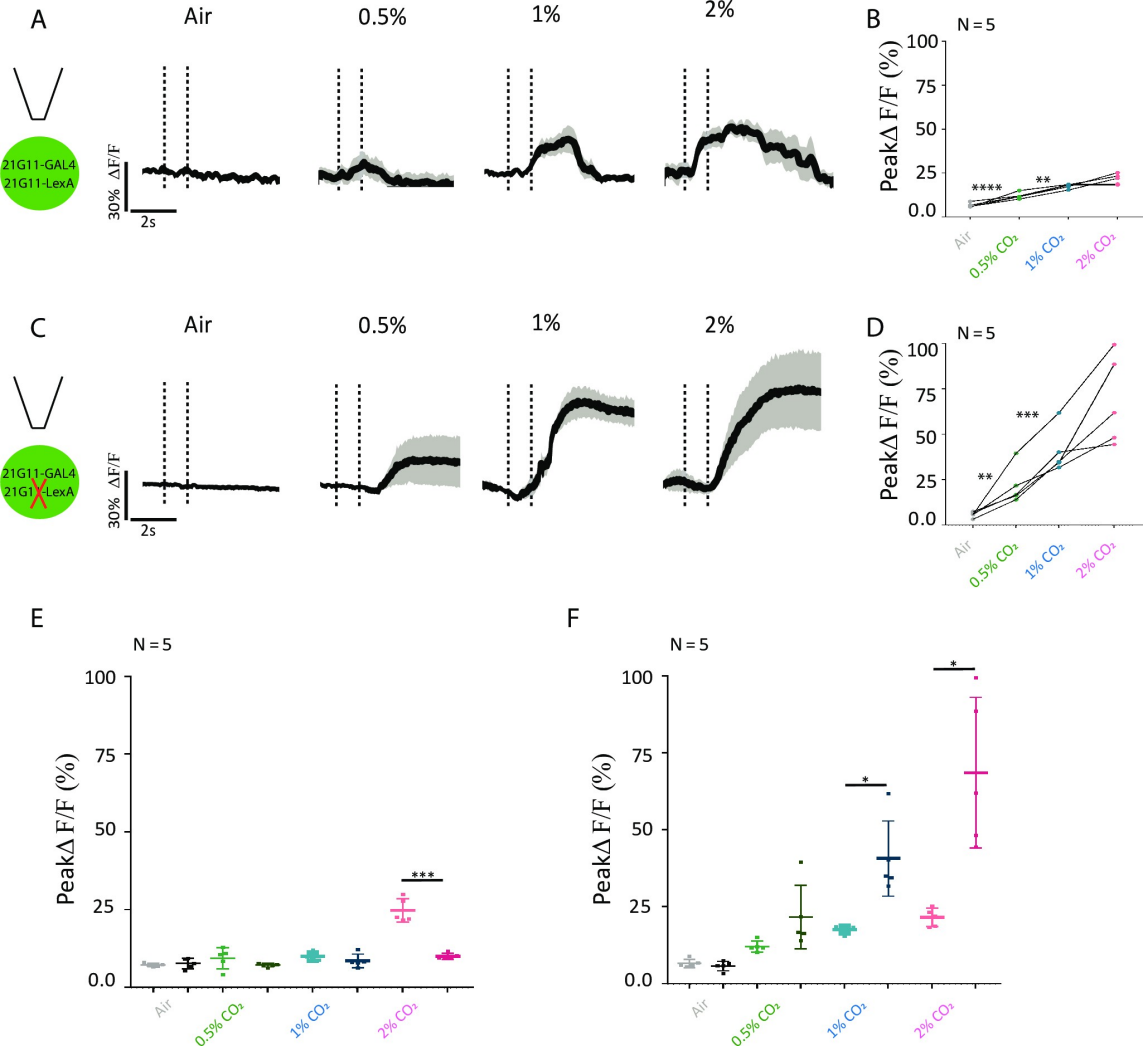
*21G11* neurons change their response profile when their *21G11-LexA* subset is silenced. (A) Schematics of the experiment and calcium response at the LH, using GCaMP6m, of *21G11* neurons to air, 0.5%, 1%, and 2% of CO_2_. (B) Peak GCaMP6m intensity change after stimulation with air, 0.5%, 1%, and 2% of CO_2_. (C) Schematics of the experiment and calcium response at the LH of *21G11* neurons to air, 0.5%, 1%, and 2% of CO_2_ while *21G11-LexA* neurons are silenced by expression of Kir2.1. (D) Peak GCaMP6m intensity change after stimulation with air, 0.5%, 1%, and 2% of CO_2_. (E–F) Peak GCaMP6m intensity change during stimulation period (E) and poststimulation period (F), with air, 0.5%, 1%, and 2% of CO_2_ for both (B) values on the left and (D) values on the right. For (A) and (C), the average time course of GCaMP6m intensity change is shown. The vertical dashed lines indicate the time of the stimulus. *p < 0.05, **p < 0.01, ****p < 0.0001. All *p* values are calculated with Wilcoxon signed-rank test. LH, lateral horn.

Next, we explored how activity in *21G11-LexA* neurons is required for the behavioral response to CO_2_ at three different concentrations when the neurons respond only to the lowest concentration (0.5% CO_2_). Since *21G11-LexA* neurons are essentially a subset of a cluster defined by expression of *21G11-GAL4*, we reasoned that perhaps subthreshold activity in *21G11-LexA* neurons influences activity of *21G11-GAL4* neurons that respond to 1% and 2% CO_2_. We silenced *21G11-LexA* neurons while recording calcium activity in *21G11-GAL4* neurons to CO_2_ stimulation (Fig 5). When *21G11-LexA* neurons are silenced, the responses within the stimulation period are all rather low, though the difference to control only reaches significance at 2% CO_2_ (Fig 5E, 0.5%: Wilcoxon signed-rank test w = −13.00, *p* = 0.2231; 1%: Wilcoxon signed-rank test w = −13.00, *p* = 0.338; 2%: Wilcoxon signed-rank test w = 13.00, *p* = 0.0003). However, a few seconds after stimulation, we observe for all concentrations a large and persistent rise in free calcium (Fig 5F, 0.5%: Wilcoxon signed-rank test w = −15.00, *p* = 0.1272; 1%: Wilcoxon signed-rank test w = 17.00, *p* = 0.0188; 2%: Wilcoxon signed-rank test w = 15.00, *p* = 0.0124). The persistence of calcium is so extended that the stimulation protocol had to be readjusted from 20 to 80 s between stimulations. Thus, the response profile changes considerably when *21G11-LexA* neurons are silenced, which could explain the change in the behavioral response. The exact nature of the interaction between subsets of *21G11* neurons still needs to be defined.

### *21G11* and *23C09* neurons are selectively involved in processing CO_2_ avoidance

**Fig 6:**
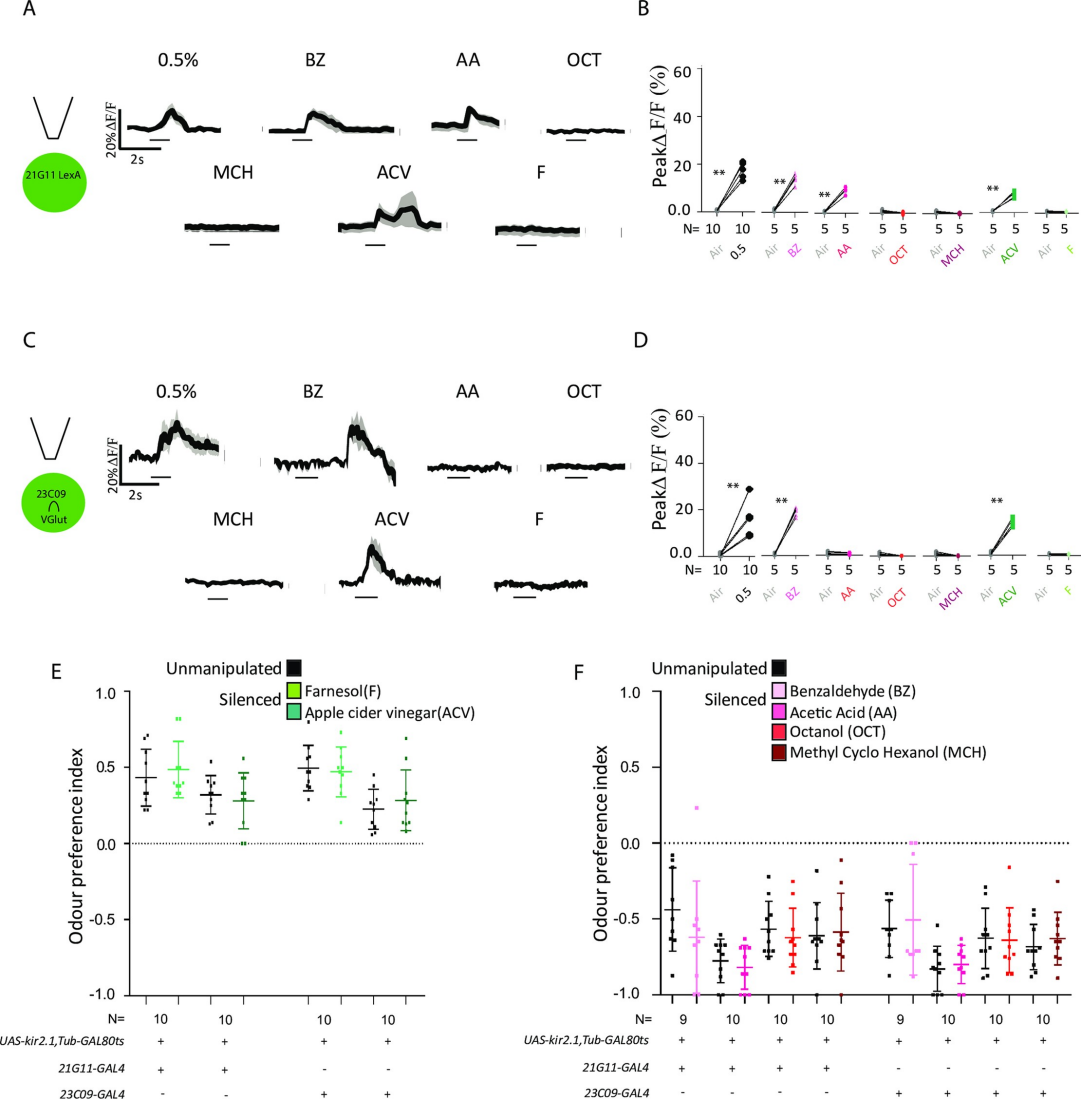
Physiological and behavioral response to attractive and repulsive compounds of *21G11* and *23C09* neurons. (A and C) Schematics of the experiment and calcium response at the LH of *21G11-LexA* and of *23C09∩VGlut* to the repulsive compounds—BZ, AA, OCT, and MCH—and the attractive compounds—F and ACV. The image shows the average time course of GCaMP6m intensity change. The black bar indicates the time of the stimulus. (B and D) Peak GCaMP6m intensity change after stimulation with 0.5% CO_2_, BZ, AA, OCT, MCH, ACV, and F. (E) T-maze response to F and ACV of *21G11* and *23C09*. (F) T-maze response to BZ, AA, OCT, and MCH of *21G11* and *23C09*. (E–F) Black dots, no heat induction of Kir2.1 expression (see materials and methods). Colored dots, heat induction of Kir2.1 expression before test. The top and bottom lines represent the first and the third quartiles. The line across the box is the median. For (B) and (D), **p < 0.01. *p* values are calculated with Wilcoxon signed-rank test. For (E) and (F), comparisons calculated with one-way ANOVA are nonsignificant. AA, acetic acid; ACV, apple cider vinegar; BZ, benzaldehyde; F, farnesol; MCH, methylcyclohexanol; OCT, octanol.

We then asked how specific the circuit we identified at the LH is. Is it generally involved in odor responses or is it involved specifically in avoidance responses? To answer this question, we first measured the calcium responses to different odors (Fig 6). To test attractive odor responses, we used farnesol (F), an attractant present in the rind of ripe citrus and processed through a single glomerulus [10], and apple cider vinegar (ACV), a complex attractive stimulus [43]. While ACV elicits a small response in both sets of neurons, F does not elicit a response in either set of neurons (Fig 6A–6D, F: Wilcoxon signed-rank test not significant; ACV: Wilcoxon signed-rank test w = 16.00, *p* = 0.0004). To test aversive responses, we used benzaldehyde (BZ), which smells of bitter almond, and acetic acid (AA), which elicits the acid-sensing response in the antennal lobe [27]. We also used octanol (OCT) and methylcyclohexanol (MCH), which are aversive at higher concentrations [44]. *21G11* neurons respond to both BZ and AA (Fig 6A and 6B, BZ: Wilcoxon signed-rank test w = 18.00, *p* = 0.0001; AA: Wilcoxon signed-rank test w = 19.00, *p* = 0.0001). *23C09∩VGlut* neurons respond only to BZ in an atypical fashion (Fig 6C and 6D). The rise in calcium concentration happens a few seconds after stimulus presentation. It is not clear what the origin of the delayed response is. It could represent rebound excitation but also could stem from a delayed response to BZ at the PN level, as it has been reported in some instances [8]. *21G11* neurons and *23C09∩VGlut* neurons do not respond to OCT and MCH (Wilcoxon signed-rank test not significant). Though the peak ΔF/F in all these responses is low, the physiological response is broad and includes responses to aversive and attractive odors. How do these physiological responses translate into a behavioral response? To address this question, we tested the requirement of activity in *23C09* or *21G11* neurons for the behavioral response to other odors. We used the original *GAL4* lines because they have broader expression. Similarly to what we did in the screen, we tested the flies using a T-maze. Also following the screen conditions, we silenced the neurons with Kir2.1 only in the adult stage. We allowed the flies to choose between air and F, ACV, AA, or BZ at 1/1000 dilution and octanol and MCH at 1/500 dilution. It was reported that there is a nonolfactory component to BZ avoidance at 1/100 dilution. We confirmed that we are not including a nonolfactory component in our experiment by testing the response of flies without olfactory organs to our working dilution of BZ (S8 Fig, Mann–Whitney, U = 45, *p* = 0.0007). We observe that activity in *23C09* or *21G11* neurons is not required either for attraction to F or ACV, indicating that activity in these LHNs is not involved in general odor responses (Fig 6E, Mann–Whitney not significant). Silencing *23C09* or *21G11* neurons also does not affect avoidance to BZ, AA, OCT, and MCH (Fig 6F, Mann–Whitney not significant). Interestingly, though the arborization at the LH of the acid-sensing PNs is very similar to the arborization of the VPNs [23], it appears that different LHNs process these responses. The results indicate that activity in *21G11* and *23C09* neurons is selectively required for the response to CO_2_.

## Discussion

In early studies, LHNs that respond to the male pheromone 11-*cis*-vaccenyl acetate were identified based on the expression of the male-specific form of the transcription factor fruitless [45,46]. One cluster of male LHNs responds specifically to the pheromone. These results opened the possibility that each odor has a cognate LHN. However, activity and anatomy analysis on larger sets of LHNs suggests a mixed model of connectivity [35,47,48]. Two very recent studies on large sets of LHNs convey important insights into LH organization [35,48]. Both studies show that genetically labeled LHNs show stereotyped responses from animal to animal. Jeanne and colleagues have photostimulated PNs at the antennal lobe while recording from 110 LHNs of 39 morphological types [48]. They find that certain combinations of glomeruli are over-represented at LHN recordings. These combinations represent an odor scene, for instance, odors relevant for egg-laying site selection rather than a single odorant. Frechter and colleagues have characterized the cellular composition of LH using a combination of anatomical analysis of genetic driver lines and whole-brain electron microscopy data [35]. They find that the LH contains over 1,300 neurons, of which 580 are LH local neurons and 830 are LH output neurons. LHNs were clustered into 150 cell types, revealing unanticipated complexity in this brain region. LHN recordings indicate that LHNs are better odor categorizers than PNs.

In this study, we used a behavioral readout to directly address the role of the LH in olfactory responses. We showed that the activity of two clusters of LHNs is required for *D*. *melanogaster* innate avoidance of CO_2_. *23C09* is a cluster of LH local neurons and *21G11* is a cluster of output neurons. Both clusters show a physiological response to CO_2_. We were able to show that *21G11-LexA*, a subset of *21G11* neurons, outputs to *23C09* within the LH. *21G11-LexA* activity in response to CO_2_ originates in the activity of *Gr21a* neurons that converge to the V-glomerulus. The response of *21G11-LexA* neurons is shaped by the activity of VPNs, although they are not directly connected. Our results define a circuit within the LH region specific for CO_2_ avoidance responses in the lower concentration range that starts at the V-glomerulus. However, the avoidance to CO_2_ is not completely abolished by silencing these neurons. This observation could have three origins: not all cells are labeled within the identified cluster; other unidentified clusters are involved; or the LH has intricate connectivity, and silencing of any LHN leads to disruption of the behavior. The latter is unlikely because we silenced and tested 32 lines labeling different LHNs, some of them innervating very large sections of the LH, and only the two lines described here consistently had an effect. Though we cannot eliminate any of the former possibilities, the lack of contact between the LHNs we identified and the VPNs that carry CO_2_ stimulus information argues for the involvement of additional neurons within the LH. In addition, a contribution from the MB cannot be excluded since MB output neuron (MBON) activity is required for naïve avoidance of odors, including CO_2_, and intrinsic MB neurons participate in the CO_2_ response when flies are starved [18,44,49].

The LH is required for the execution of learned aversion [50]. Hence, the roles of the LH and MB centers are not as segregated as previously thought. They must be connected to coordinate the innate, the context-dependent, and the learned responses. There are at least two LH outputs to the CO_2_ response. *21G11* neurons output within the LH and connect to the SIP. The SIP is an area highly innervated by MBON terminals, suggesting a location for integration of LH and MB output [25,51]. A preprint published while this manuscript was under revision suggests that presynaptic labeling (as well as postsynaptic labeling) in dendrites of LH output neurons, such as we observe in *21G11* neurons, is a common occurrence and estimates that one third of LH projections converge to sites of MB outputs [52].

*21G11* and *23C09* show specificity to CO_2_ behavioral response. Activity in these neurons is not required for the T-maze response to other four aversive odors. The most logical organization for a center that processes innate behavioral responses is that inputs that elicit a particular behavioral outcome (such as avoidance) converge into a particular region. However, our work indicates that not all avoidance responses are processed equally. Still, it is possible that the CO_2_ response converges with the response to other aversive stimuli downstream of *23C09* neurons. A candidate region of the LH tuned to repulsive odors has been described [53].

Our findings revealed a disparity between activity of the LHNs we identified and the behavioral requirement for a few of the tested odors. The result indicates that not all activity in these LHNs will drive the behavior. This means that the LH is more complex than previously anticipated. We speculate that since innate responses can be flexible, in a different context, the activity here revealed will shape the behavioral response to other odors.

A vast search has identified compounds that either increase or decrease the activity of the CO_2_ receptors in mosquitoes [54]. A later study in the fly revealed that odors increasing activity of *Gr21-Gr63a* receptor neurons generate avoidance responses and odors decreasing receptor activity generate attraction responses, indicating that the CO_2_ receptor pathway has a strong weight in establishing odor valence [55]. Further work should elucidate how the LHNs identified here contribute to these responses.

In summary, we demonstrated a role of the LH in an innate behavioral response. A preprint posted while this manuscript was under revision confirms this conclusion, looking at egg-laying aversion [56]. Moving forward, it will be interesting to explore whether a similar organization at the LH is used to generate responses to other odors and how the response is coordinated with the MB.

## Materials and methods

### Contact for reagent and resource sharing

Further information and requests for resources and reagents should be directed to and will be fulfilled by the lead contact, Maria Luísa Vasconcelos (maria.vasconcelos@neuro.fchampalimaud.org).

### Experimental model and subject details

Flies were maintained on standard cornmeal–agar medium at 18°C or 25°C and 70% relative humidity under a 12 h light/dark cycle. Fly strains used were as follows: *UAS-Kir2*.*1* [33]; *UAS>stop>Kir2*.*1* [57]; *UAS>stop>mCD8GFP* [58]; *tub-GAL80*^*TS*^ [34]; *UAS-Chrimson* [42]; *UAS-GCaMP6m* [40]; *UAS-Dscam17*.*1-GFP* [38]; *UAS-syt-HA* [39]; *UAS-CD4-GFP*_*1-10*_ [41]; *UAS-CD8-GFP* [59]; *VGlut-DBD* [37]; *UAS-myr-tdTomato* [60]; *LexAop-Kir2*.*1* (provided by Barry Dickson) [61]; *LexAop-Chrimson* [62]; *LexAop-GCaMP6m* [40]; *LexAop-syt-HA* [63]; *LexAop-CD4-GFP*_*11*_ [41]; *LexAop-mCD2-GFP* [64]. We used the following *GAL4* lines: *21G11*, *23C09*, *84A06*, *65D12*, *19B07*, *13A11*, *30A10*, *37G11*, *33E01*, *93D02*, *93D05*, *41F11*, *85C07*, *64B02*, *36E10*, *30H02*, *25B07*, *36G09*, *23F06*, *54G12*, *16C09*, *13A07*, *22B02*, *29F04*, *16C06*, *82E01*, *25A01*, *84G12*, *25G10*, *26C12*, *20C09*, *20B0*, *53A05*, *41C05* from the Janelia farm collection [31,32], *Gr21a-GAL4* [65], and *otdFLP* [66]. *21G11-LexA* and *23C09-AD* were generated for this work (see Method details).

### Genotypes per figure

**Fig 1.** Panel A

*w^1118^*; *; 21G11-GAL4/+*

*w^1118^*;*UAS-Kir2.1,tub-Gal80^TS^/+;21G11-GAL4/+*

*w^1118^*; *; 23C09-GAL4/+*

*w^1118^*;*UAS-Kir2.1,tub-Gal80^TS^/+;23C09-GAL4/+*

Panel B

*w^1118^*;*LexAop-Kir2.1/+;+*

*w^118^*; *21G11-LexA/+;21G11-LexA/+*

*w^1118^*; *21G11-LexA/LexAop-Kir2.1;21G11-LexA/+*

Panel C

*w^1118^*;*UAS-Kir2.1,tub-Gal80^TS^/+;+*

*w^1118^*; *23C09-AD,VGlut-DBD/+;+*

*w^1118^*;*UAS-Kir2.1,tub-Gal80^TS^/23C09-AD,VGlut-DBD;+*

Panel D

*w^1118^*; *;21G11-GAL4/UAS-mCD8-GFP*

*w^1118^;LexAop-mCD2-GFP/+*;*21G11-LexA/+*

Panel E

*w^1118^*; *;23C09-GAL4/UAS-mCD8-GFP*

*w^1118^;23C09-AD/VGlut-DBD*;*UAS-mCD8-GFP/+*

Panels F and G

*w^1118^*;*UAS-Dscam-17.1-GFP/+;21G11-GAL4/+*

*w^1118^*;*UAS-syt-HA/+;21G11-GAL4/+*

Panel H

*w^1118^;21G11-LexA/LexAop-CD2-GFP*;*UAS-myr-tdTomato/53A05-GAL4*

*w^1118^*; *21G11-LexA/ LexAop-CD2-GFP*;*UAS-myr-tdTomato /41C05-GAL4*

Panel I

*w^1118^*; *23C09-AD/VGlut-DBD*;*UAS-mCD8-GFP/53A05-GAL4*

*w^1118^*; *23C09-AD/VGlut-DBD*;*UAS-mCD8-GFP/41C05-GAL4*

**Fig 2.** Panels A and B

*w^1118^*;*UAS-GCaMP6m/+;21G11-GAL4/+*

Panels C and D

*w^1118^*;*21G11-LexA/+;21G11-LexA/LexAop-GCaMP6m*

Panels E and F

*w^1118^;23C09-AD/VGlut-DBD*;*UASGCaMP6m/+*

**Fig 3.** Panel A

*w^1118^;LexAop-CD4-GFP_11_/21G11-LexA*;*UAS-mCD4-GFP_1-10_/23C09-GAL4*

Panels B, C, and D

*w^1118^*;*21G11-LexA,LexAop-Kir2.1/23C09-AD,VGlut-DBD;21G11-LexA/UAS-GCaMP6m*

Panels E and F

*w^1118^*;*21G11-LexA,LexAop-Chrimson/23C09-AD,VGlut-DBD;21G11-LexA/UASGCaMP6m*

Panels G and H

*w^1118^*;*21G11-LexA/23C09-AD,VGlut-DBD;21G11-LexA,LexAop-GCaMP6m/UAS-Kir2.1*

Panel I

*w^1118^*;*21G11-LexA/23C09-AD,VGlut-DBD;21G11-LexA,LexAop-GCaMP6m/UAS-Chrimson*

**Fig 4.** Panels A and B

*w^1118^*;*21G11-LexA,LexAop-GCaMP6m/Gr21a-GAL4;21G11-LexA/UAS-Kir2.1*

Panels C and D

*w^1118^*;*21G11-LexA,LexAop-GCaMP6m/Gr21a-GAL4;21G11-LexA/UAS-Chrimson*

Panels E and F

*w^1118^*;*21G11-LexA/UAS-Chrimson;21G11-LexA,LexAop-GCaMP6m/41C05-GAL4*

Panels G and H

*w^1118^*;*21G11-LexA/UAS-Chrimson;21G11-LexA,LexAop-GCaMP6m/53A05-GAL4*

**Fig 5.** Panels A and B

*w^1118^*;*21G11-LexA/UAS-GCaMP6m;21G11-LexA/21G11-GAL4*

Panels C and D

*w^1118^*;*21G11-LexA,LexAop-Kir2.1/UAS-GCaMP6m;21G11-LexA/21G11-GAL4*

Panel E

*w^1118^*;*21G11-LexA/UAS-GCaMP6m;21G11-LexA/21G11-GAL4*

*w^1118^*;*21G11-LexA,LexAop-Kir2.1/UAS-GCaMP6m;21G11-LexA/21G11-GAL4*

**Fig 6.** Panels A and B

*w^1118^*;*21G11-LexA/+;21G11-LexA/LexAop-GCaMP6m*

Panels C and D

*w^1118^;23C09-AD/VGlut-DBD*;*UASGCaMP6m/+*

Panels E and F

*w^1118^*;*UAS-Kir2.1,tub-Gal80^TS^/+;21G11-GAL4/+*

*w^1118^*;*UAS-Kir2.1,tub-Gal80^TS^/+;23C09-GAL4/+*

**S1 Fig.** Panel A

*w^1118^*;*UAS-Kir2.1,tub-Gal80^TS^/+;84A06-GAL4/+*

*w^1118^*;*UAS-Kir2.1,tub-Gal80^TS^/+;65D12-GAL4/+*

*w^1118^*;*UAS-Kir2.1,tub-Gal80^TS^/+;21G11-GAL4/+*

*w^1118^*;*UAS-Kir2.1,tub-Gal80^TS^/+;23C09-GAL4/+*

*w^1118^*;*UAS-Kir2.1,tub-Gal80^TS^/+;19B07-GAL4/+*

*w^1118^*;*UAS-Kir2.1,tub-Gal80^TS^/+;13A11-GAL4/+*

*w^1118^*;*UAS-Kir2.1,tub-Gal80^TS^/+;30A10-GAL4/+*

*w^1118^*;*UAS-Kir2.1,tub-Gal80^TS^/+;37G11-GAL4/+*

*w^1118^*;*UAS-Kir2.1,tub-Gal80^TS^/+;33E01-GAL4/+*

*w^1118^*;*UAS-Kir2.1,tub-Gal80^TS^/+;93D02-GAL4/+*

*w^1118^*;*UAS-Kir2.1,tub-Gal80^TS^/+;93D05-GAL4/+*

*w^1118^*;*UAS-Kir2.1,tub-Gal80^TS^/+;41F11-GAL4/+*

*w^1118^*;*UAS-Kir2.1,tub-Gal80^TS^/+;85C07-GAL4/+*

*w^1118^*;*UAS-Kir2.1,tub-Gal80^TS^/+;64B02-GAL4/+*

*w^1118^*;*UAS-Kir2.1,tub-Gal80^TS^/+;36E10-GAL4/+*

*w^1118^*;*UAS-Kir2.1,tub-Gal80^TS^/+;30H02-GAL4/+*

*w^1118^*;*UAS-Kir2.1,tub-Gal80^TS^/+;25B07-GAL4/+*

*w^1118^*;*UAS-Kir2.1,tub-Gal80^TS^/+;36G09-GAL4/+*

*w^1118^*;*UAS-Kir2.1,tub-Gal80^TS^/+;23F06 GAL4/+*

*w^1118^*;*UAS-Kir2.1,tub-Gal80^TS^/+;54G12-GAL4/+*

*w^1118^*;*UAS-Kir2.1,tub-Gal80^TS^/+;16C09-GAL4/+*

*w^1118^*;*UAS-Kir2.1,tub-Gal80^TS^/+;13A07-GAL4/+*

*w^1118^*;*UAS-Kir2.1,tub-Gal80^TS^/+;22B02-GAL4/+*

*w^1118^*;*UAS-Kir2.1,tub-Gal80^TS^/+;29F04-GAL4/+*

*w^1118^*;*UAS-Kir2.1,tub-Gal80^TS^/+;16C06-GAL4/+*

*w^1118^*;*UAS-Kir2.1,tub-Gal80^TS^/+;82E01-GAL4/+*

*w^1118^*;*UAS-Kir2.1,tub-Gal80^TS^/+;25A01-GAL4/+*

*w^1118^*;*UAS-Kir2.1,tub-Gal80^TS^/+;84G12-GAL4/+*

*w^1118^*;*UAS-Kir2.1,tub-Gal80^TS^/+;25G10-GAL4/+*

*w^1118^*;*UAS-Kir2.1,tub-Gal80^TS^/+;26C12-GAL4/+*

*w^1118^*;*UAS-Kir2.1,tub-Gal80^TS^/+;20C09-GAL4/+*

*w^1118^*;*UAS-Kir2.1,tub-Gal80^TS^/+;20B07-GAL4/+*

Panel B

*w^1118^*;*UAS-Kir2.1,tub-Gal80^TS^/+;65D12-GAL4/+*

*w^1118^*;*UAS-Kir2.1,tub-Gal80^TS^/+;21G11-GAL4/+*

*w^1118^*;*UAS-Kir2.1,tub-Gal80^TS^/+;23C09-GAL4/+*

*w^1118^*;*UAS-Kir2.1,tub-Gal80^TS^/+;13A11-GAL4/+*

*w^1118^*;*UAS-Kir2.1,tub-Gal80^TS^/+;33E01-GAL4/+*

*w^1118^*;*UAS-Kir2.1,tub-Gal80^TS^/+;85C07-GAL4/+*

*w^1118^*;*UAS-Kir2.1,tub-Gal80^TS^/+;36G09-GAL4/+*

*w^1118^*;*UAS-Kir2.1,tub-Gal80^TS^/+;16C09-GAL4/+*

**S2 Fig.** Panel A

*UAS>stop>Kir2.1/ w^118^*;*otdFLP/+;+*

*w^118^*; *;21G11-GAL4/+*

*w^1118^*; *;23C09-GAL4/+*

*UAS>stop>Kir2.1/ w^118^*;*otdFLP/+;21G11-GAL4/+*

*UAS>stop>Kir2.1/ w^118^*;*otdFLP/+;23C09-GAL4/+*

Panel B

*w^1118^*; *otdFLP /+;21G11-GAL4/ UAS>stop>mCD8-GFP*

*w^1118^*; *otdFLP /+;23C09-GAL4/ UAS>stop>mCD8-GFP*

Panel C

*w^1118^*;*UAS-Kir2.1,tub-Gal80^TS^/+;21G11-GAL4/+*

*w^1118^*;*UAS-Kir2.1,tub-Gal80^TS^/+;23C09-GAL4/+*

*w^1118^*;*UAS-Kir2.1,tub-Gal80^TS^/+;21G11-GAL4/23C09-GAL4*

Panel D

*w^118^*; *;21G11-GAL4/+*

*w^1118^*;*UAS-Kir2.1,tub-Gal80^TS^/+;21G11-GAL4/+w^1118^*; *;23C09-GAL4/+*

*w^1118^*;*UAS-Kir2.1,tub-Gal80^TS^/+;23C09-GAL4/+*

*w^1118^*; *;21G11-GAL4/23C09-GAL4*

*w^1118^*;*UAS-Kir2.1,tub-Gal80^TS^/+;21G11-GAL4/23C09-GAL4*

**S3 Fig**.

*w^1118^*; *21G11-LexA /LexAop-mCD2-GFP;21G11-LexA/21G11-GAL4,UAS-myr-tdTomato*

**S4 Fig.** Panel A

*w^1118^*;*LexAop-mCD2-GFP/+;21G11-LexA/+*

Panel B

*w^1118^;23C09-AD/VGlut-DBD*;*UAS-mCD8-GFP/+*

Panel C

*w^1118^*; *;21G11-GAL4/UAS-mCD8-GFP*

*w^1118^*;*LexAop-mCD2-GFP/+;21G11-LexA/+*

Panel D

*w^1118^*; *;23C09-GAL4/UAS-mCD8-GFP*

*w^1118^;23C09-AD/VGlut-DBD*;*UAS-mCD8-GFP/+*

**S5 Fig.** Panel A

*w^1118^*;*LexAop-syt-HA/+;21G11-LexA/+*

Panel B

*w^1118^*;*UAS-Dscam-17.1-GFP/23C09-AD,VGlut-DBD;+*

*w^1118^*;*UAS-syt-HA/23C09-AD,VGlut-DBD;+*

**S6 Fig.** Panels A and B

*w^1118^*;*21G11-LexA,LexAop-Kir2.1/+;21G11-LexA/LexAop-GCaMP6m*

Panels C and D

*w^1118^*;*21G11-LexA,LexAop-Chrimson/+;21G11-LexA/LexAop-GCaMP6m*

**S7 Fig.** Panels A and B

*w^1118^;23C09-AD,VGlut-DBD/+*;*UASGCaMP6m/UAS-Kir2.1*

Panels C and D

*w^1118^;23C09-AD,VGlut-DBD/+*;*UASGCaMP6m/UAS-Chrimson*

**S8 Fig**.

*w^1118^*;*UAS-Kir2.1,tub-Gal80^TS^/+;+*

### Method details

#### Generating transgenic flies

For the establishment of the *21G11-LexA* lines, we used the Gateway System. The *LexA* vector used was purchased from Addgene (plasmid #26230; Watertown, MA, USA). We carried out a genomic PCR with the primers (5′ to 3′) F-GGGGACAAGTTTGTACAAAAAAGCAGGCTTCGCGCAGCACGTGAAGAACAAGGC and R-GGGGACCACTTTGTACAAGAAAGCTGGGTCATGGCAACGTACTTCCAGTCCTCT. The fragment was inserted in a pDONR221 vector and then recombined into the pBPnlsLexA::p65Uw vector (Addgene, plasmid #26230). To construct the *23C09AD* line, we use the fragment in a pDON that was kindly provided by the Rubin Lab. We recombined it as described above. *21G11-LexA* was injected into attp40 and attp2 flies. *23C09-AD* was injected into attp40 flies.

#### Immunostaining

Adult fly brains were dissected, fixed, and stained using standard protocols. Briefly, tissue was dissected in phosphate-buffered saline (PBS), fixed in 4% PFA in PBL (PBS and 0.12M lysine) for 30 min at room temperature, washed 3× for 5 min in PBT (PBS and 0.5% Triton X-100), and blocked for 15 minutes in 10% normal goat serum in PBT (Sigma Aldrich, cat# G9023; St. Louis, MO, USA). Samples were then incubated with primary antibodies for 72 h at 4°C. After incubation, they were washed 3× for 10 min in PBT and incubated with secondary antibodies for 72 h at 4°C. Finally, the samples were washed 3× for 10 min in PBT and mounted in Vectashield (Vector Laboratories, cat# H-1000; Burlingame, CA, USA). As primary antibodies, we used: rabbit anti-GFP (1:2000, Molecular Probes, cat# 11122; Eugene, OR, USA), chicken anti-GFP (1:2000, Molecular Probes, cat# A10262), rabbit anti-DsRed (1:2000, Molecular Probes, cat# 710530), and mouse anti-nc82 (1:10, Developmental Studies Hybridoma Bank, University of Iowa, Iowa City, IA, USA). The secondary antibodies used were anti-rabbit or anti-chicken IgG conjugated to Alexa 488, anti-mouse or anti-rabitt IgG conjugated to Alexa 594, and anti-mouse IgG conjugated to Alexa 405. All microscopy of immunostainings was performed with a Zeiss LSM 710 confocal microscope (Zeiss, Oberkochen, Germany). Images were processed with ImageJ.

#### Behavioral experiments

Neuronal silencing: Flies were kept at 18°C for 8 to 16 d. When using the *UASKir2*.*1*,*TubGAL80*^*ts*^, tester flies were placed at 30°C 24 h before the experiment, while control flies were always kept at 18°C. On the day of the experiment, both tester and control flies were transferred to 25°C where we quantified their response in a T-maze [67]. When using the *LexAopKir2*.*1* flies, both tester and control flies were always kept at 25°C. We quantified flies’ response to air, three concentrations of CO_2_ (0.5%, 1%, and 2%), and two known attractive (ACV and F) and four known repulsive compounds (BZ, AA, OCT, and MCH). To obtain the CO_2_ concentrations, we mixed bottled synthetic air with bottled CO_2_ (Linde, Danbury, CT, USA). The flow rate used was of 0.12 l per min. All other compounds were diluted 1:1000 in paraffin oil (Sigma Aldrich) with the exception of OCT and MCH, for which the dilution was 1:500 in paraffin oil. To test for starvation, adult flies were removed from vials with food and placed in vials with humidified paper 24 h prior to the experiment. To test for the nonolfactory component to BZ avoidance, the olfactory organs were removed manually (antennae and maxillary palps) 24 h before the experiment. For all experiments, flies were tested in groups of 20 individuals. Flies were placed on the T-maze elevator and dropped to the choice area, where they were given 45 seconds to choose an arm. To control whether the T-maze was balanced, we tested flies to air on both sides. For control and tester flies, one arm of the T-maze released air, while the other arm released the testing compound. After the experiment, flies were counted, and the odor preference index was calculated by subtracting the number of flies on the air side from the number of flies on the other side and dividing it with the total number of flies.

#### Calcium imaging experiments

Preparation: For all calcium imaging experiments, flies expressed the calcium indicator *GCaMP6m*. The preparation was based on walking behavior preparation [68] but without the ball. To image the LH in an in vivo preparation, we glued the fly head to a microscope base (Scientifica, Uckfield, UK) with bee’s wax (Sigma Aldrich). We then opened a window that corresponded to half of the fly brain and removed all fat and trachea. We made sure that both antennae were untouched and healthy.

Microscopy: We used an Ultima two-photon laser-scanning microscope from Prairie Technologies (now Bruker; Billerica, MA, USA) and a Coherent Chameleon XR laser (Coherent, Santa Clara, CA, USA). All images were acquired every 0.2 ms with an Olympus BX61 microscope (Olympus, Tokyo, Japan) equipped with a 40 × 0.8 NA objective. The image was zoomed to allow the selection of a region of interest (ROI) in the LH where the stimulation was performed. To measure the fluorescent intensity at the LH, we used ImageJ. In the image recorded at the time of the experiment, we delineated by hand and around the observable innervation of the LH a second ROI. The resulting time trace from the second ROI was used for further analysis. To calculate the normalized change in the relative fluorescence intensity, we used ΔF/F = 100(F_n_ − F_0_)/F_0_, where F_n_ is the *n*th frame after stimulation and F_0_ is the average basal fluorescence before the stimulation. Images with visible rhythmic movements of the animal were discarded.

Olfactory stimulation: For olfactory stimulation, a custom-made delivery system consisting of a four-way solenoid valve (Parker Hannifin, Cleveland, OH, USA) connected to a peristaltic pump (Ismatec, Wertheim, Germany) creating a continuous airstream (1800 ml/min) that was delivered to the antennae with chemically inert tubing (Ismatec). The valve stimulation was commanded through the PrairieView software. For the CO_2_ stimulation, dilutions were placed in Tedlar gas sampling bags (#24634, Sigma Aldrich) that were then connected to the valve. For stimulation with other compounds dilutions were made in glass vials with rubber taps (Thermo Fisher Scientific, Waltham, MA, USA). At the rubber tap, we inserted two venofix needles (B. Braun, Melsungen, Germany): one to connect the vial to the valve, the other to connect the vial to the air in the room for airflow in the vial. We set up the system so that when a stimulus is triggered, the odor dilutions replace only 50% of the airflow in order to minimize the turbulence. For this reason, all dilutions were prepared to double of the desired concentrations. In all experiments, stimuli were delivered for one second. The interval between stimuli was of 20 s for all experiments except for Fig 5C and 5D, in which the recovery time proved to be longer. For this experiment, the interval was set at 80 s. To control for calcium changes with the airflow, all stimulations were done twice. In addition, we performed experiments both in and outside the LH and imaged the LH in neurons with both *Kir2*.*1* and *GCaMP6m*. No interference from the airflow in the calcium response was ever observed.

Neuronal activation: For the neuronal activation, we used 8 LEDs of 720 nm wavelength in a custom-made ring placed beneath the stage that surrounded the fly. The delivery of light to the fly was commanded through the PrairieView software. The stimulus was delivered for one second at 5-Hz and 40-ms pulses.

Neuronal silencing: For the neuronal silencing experiments, all flies expressed *Kir2*.*1*.

#### Quantification and statistical analysis

All behavioral data was statistically analyzed by one-way analysis of variance and a Sidak’s multiple comparisons test. For all imaging data, a Wilcoxon signed-rank test comparison was performed. For all analysis and statistical tests, we used the GraphPad Prism Software version 6.0 (GraphPad Software).

The numerical data used in all figures are included in S1 Data.

## Supporting information

S1 Fig Inhibitory screen of 32 lines labeling LHNs to CO_2_ response. (A) T-maze response to 0.5% CO_2_ of 32 fly lines from the Janelia farm collection chosen by their innervation at the LH. Avoidance responses to CO_2_ are significantly reduced in eight lines (*84A06*, *65D12*, *21G11*, *23C09*, *33E01*, *85C07*, *36G09*, and *16C09*) when silenced with *UAS-Kir2*.*1*,*Tub-GAL80*^*ts*^. (A–B) The top and bottom lines represent the first and the third quartiles. The line across the box is the median. (B) Retest of T-maze response to 0.5% CO_2_ of the eight fly lines that showed reduced avoidance on (A). Avoidance responses to CO_2_ are significantly reduced in three lines (*65D12-GAL4*, *21G11-GAL4*, and *23C09-GAL4*) when silenced with *UAS-Kir2*.*1*,*Tub-GAL80*^*ts*^. *p < 0.05. All *p* values are calculated with multiple *t* test corrected with the Holm–Sidak method. LH, lateral horn; LHN, LH neuron.
(TIF)

S2 Fig Silencing brain neurons only, both sets of neurons or starvation do not change the effect of silencing the lines in response to CO_2_. (A) T-maze response to 0.5%, 1%, and 2% CO_2_ of *21G11* and *23C09* with Kir2.1 expression restricted to the brain using *UAS>stop>Kir2*.*1;otdFLP*. Post hoc two-way ANOVA comparing behavioral response to different concentrations reveals no significance. (B) Brain and VNC expression of *21G11-GAL4* and of *23C09-GAL4* intersected with otdFLP. (C) T-maze response to 1% CO_2_ of starved flies of *21G11* and *23C09*. The box represents the first and the third quartiles, and the whiskers the 10th and 90th percentiles. The line across the box is the median. N = 10. All *p* values are calculated via one-way ANOVA. (D) Retest of T-maze response to 1% CO_2_ of same lines as Fig 1A plus flies with all genetic elements combined so that neurons of both line *21G11* and *23C09* are manipulated. Post hoc two-way ANOVA comparing behavioral response of individual and combined expressions both for control and test samples reveals no significance. otdFLP, orthodenticle-flipase; VNC, ventral nerve cord.
(TIF)

S3 Fig Overlaid expression of *21G11-LexA* and *21G11*. LH cells of *21G11-LexA* (red) and *21G11-GAL4* (green) and the merge of both. Scale bar = 10 μm. LH, lateral horn.
(TIF)

S4 Fig Detailed expression of *21G11-LexA, 23C09∩VGlut*, and VNC expression for *21G11, 23C09, 21G11-LexA*, and *23C09∩VGlut*. (A and B) Brain expression every 10 μm of *21G11-LexA* (A) and *23C09∩VGlut* (B) lines. (C) VNC expression of *21G11* and *21G11-LexA*. (D) VNC expression of *23C09* and *23C09∩VGlut*. For all images, green = GFP, magenta = nc82. GFP, green fluorescent protein; nc82, monoclonal antibody to Bruchpilot; VNC, ventral nerve cord.
(TIF)

S5 Fig Dscam expression of LH *23C09*∩*VGlut* and syt-HA expression of LH *23C09*∩*VGlut* and LH and SIP of *21G11-LexA*. (A) *Syt-HA* expression in the LH and SIP of *21G11-LexA* (green). The circle highlights the LH. The vertical lobe of the MB is drawn to facilitate visualization of the adjacent SIP. (B) Dscam17.1-GFP and syt-HA expression in the LH of *23C09∩VGlut* (green). The circle highlights the LH. Scale bar = 10 μm. d, dorsal; Dscam17.1, Down syndrome cell adhesion molecule with isoform 1 of the transmembrane domain; GFP, green fluorescent protein; HA, hemagglutinin; l, lateral; LH, lateral horn; m, medial; MB, mushroom body; SIP, superior intermediate protocerebrum; syt, synaptotagmin; v, ventral.
(TIF)

S6 Fig Kir2.1 expression abolishes calcium response and 720 nm light activates Chrimson in *21G11-LexA* neurons. (A and B) Schematics of the experiment and calcium response at the LH, using GCaMP6m, of *21G11-LexA* neurons to air, 0.5%, 1%, and 2% of CO_2_ while *21G11-LexA* neurons are silenced by expression of Kir2.1. (C) Schematics of the experiment and LH activity of *21G11-LexA* upon activation of *21G11-LexA* neurons, expressing Chrimson, with 720 nm light. (D) Peak GCaMP6m intensity change upon activation. For (C), the average time course of GCaMP6m intensity change is shown. The black bar indicates the time of the stimulus. *p < 0.05. All *p* values are calculated with Wilcoxon signed-rank test. LH, lateral horn.
(TIF)

S7 Fig Kir2.1 expression abolishes calcium response and 720 nm light activates Chrimson in *23C09*∩*VGlut* neurons. (A and B) Schematics of the experiment and calcium response at the LH, using GCaMP6m, of *23C09∩VGlut* neurons to air, 0.5%, 1% and 2% of CO_2_, while *23C09∩VGlut* neurons are silenced by expression of Kir2.1. (C) Schematics of the experiment and LH activity of *23C09∩VGlut* upon activation of *23C09∩VGlut* neurons, expressing Chrimson, with 720 nm light. (D) Peak GCaMP6m intensity change upon activation. For (C), the average time course of GCaMP6m intensity change is shown. The black bar indicates the time of the stimulus. *p < 0.05. All *p* values are calculated with Wilcoxon signed-rank test. LH, lateral horn.
(TIF)

S8 Fig Response to BZ is olfactory. T-maze response to benzaldehyde at 1:1000 dilution (BZ). White box, flies with olfactory organs. Gray box, flies without olfactory organs. The box represents the first and the third quartiles, and the whiskers the 10th and 90th percentiles. The line across the box is the median. N = 10. Error bars indicate ±SEM ****p<0.0001. *p* values are calculated with Wilcoxon signed-rank test. BZ, benzaldehyde; SEM, standard error of the mean.
(TIF)

S1 Data (XLSX)

## Abbreviations

AA: acetic acid
ACV: apple cider vinegar
BZ: benzaldehyde
CCU: Champalimaud Centre for the Unknown
Dscam17.1: Down syndrome cell adhesion molecule with isoform 1 of the transmembrane domain
F: farnesol
GFP: green fluorescent protein
Gr: gustatory receptor
GRASP: GFP reconstitution across synaptic partners
HA: hemagglutinin
IR: ionotropic receptor
LH: lateral horn
LHN: LH neuron
LHON: LH output neuron
MB: mushroom body
MBON: MB output neuron
MCH: methylcyclohexanol
nc82: monoclonal antibody to Bruchpilot
OCT: octanol
PBS: phosphate-buffered saline
PN: projection neuron
PV: posterior ventral
ROI: region of interest
SIP: superior intermediate protocerebrum
VNC: ventral nerve cord
VPN: V-glomerulus–innervating PN
