# Supplementary figures and images for: Avoidance response to CO2 in the lateral horn
Source: PLoS Biol. 2019 Jan 17;17(1):e2006749. doi: 10.1371/journal.pbio.2006749 (PMC6336243; doi:10.1371/journal.pbio.2006749)

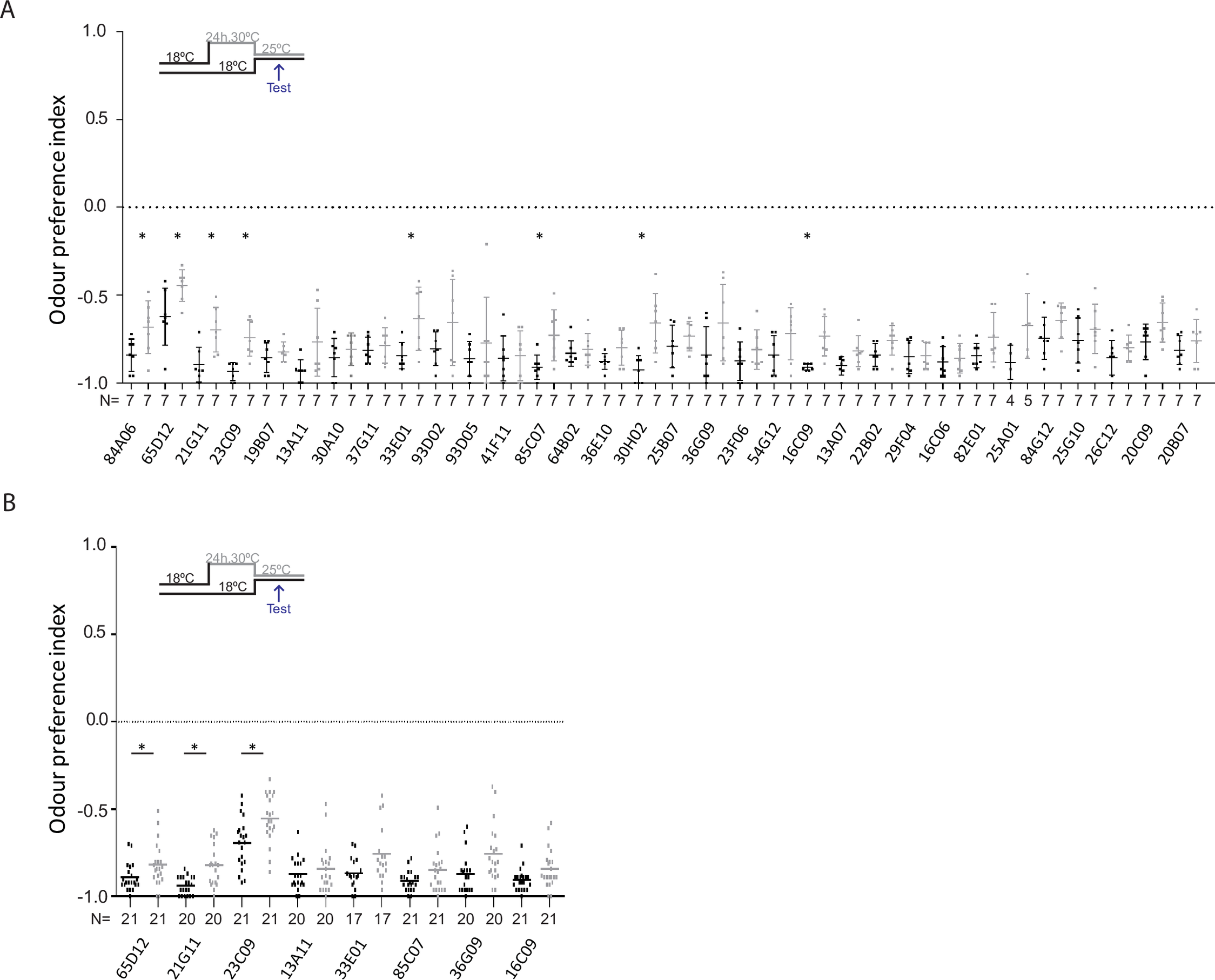

Supplement: S1 Fig — (A) T-maze response to 0.5% CO2 of 32 fly lines from the Janelia farm collection chosen by their innervation at the LH. Avoidance responses to CO2 are significantly reduced in eight lines (84A06, 65D12, 21G11, 23C09, 33E01, 85C07, 36G09, and 16C09) when silenced with UAS-Kir2.1,Tub-GAL80ts. (A–B) The top and bottom lines represent the first and the third quartiles. The line across the box is the median. (B) Retest of T-maze response to 0.5% CO2 of the eight fly lines that showed reduced avoidance on (A). Avoidance responses to CO2 are significantly reduced in three lines (65D12-GAL4, 21G11-GAL4, and 23C09-GAL4) when silenced with UAS-Kir2.1,Tub-GAL80ts. *p < 0.05. All p values are calculated with multiple t test corrected with the Holm–Sidak method. LH, lateral horn; LHN, LH neuron. (TIF) [file pbio.2006749.s001.tif]

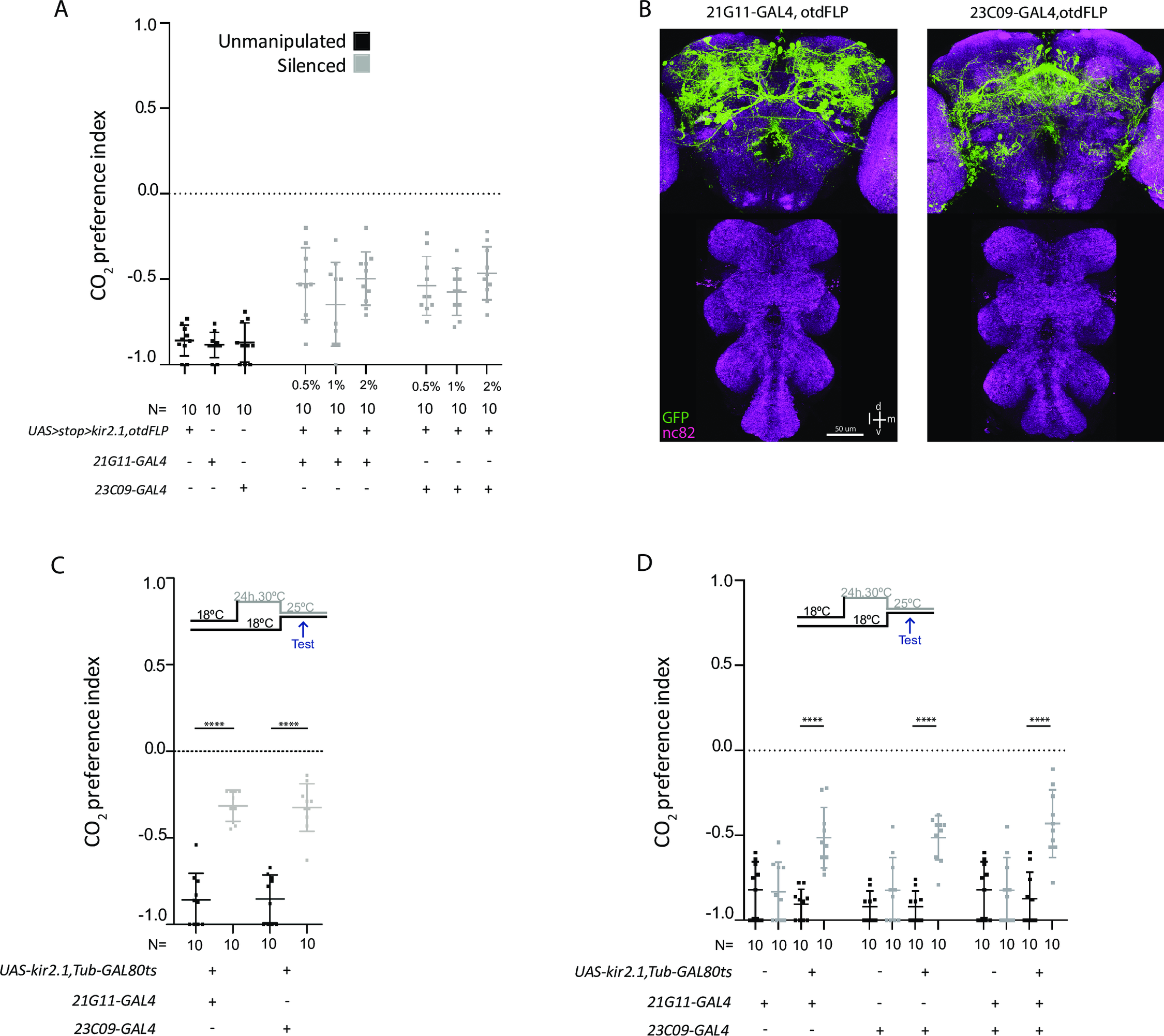

Supplement: S2 Fig — (A) T-maze response to 0.5%, 1%, and 2% CO2 of 21G11 and 23C09 with Kir2.1 expression restricted to the brain using UAS>stop>Kir2.1;otdFLP. Post hoc two-way ANOVA comparing behavioral response to different concentrations reveals no significance. (B) Brain and VNC expression of 21G11-GAL4 and of 23C09-GAL4 intersected with otdFLP. (C) T-maze response to 1% CO2 of starved flies of 21G11 and 23C09. The box represents the first and the third quartiles, and the whiskers the 10th and 90th percentiles. The line across the box is the median. N = 10. All p values are calculated via one-way ANOVA. (D) Retest of T-maze response to 1% CO2 of same lines as Fig 1A plus flies with all genetic elements combined so that neurons of both line 21G11 and 23C09 are manipulated. Post hoc two-way ANOVA comparing behavioral response of individual and combined expressions both for control and test samples reveals no significance. otdFLP, orthodenticle-flipase; VNC, ventral nerve cord. (TIF) [file pbio.2006749.s002.tif]

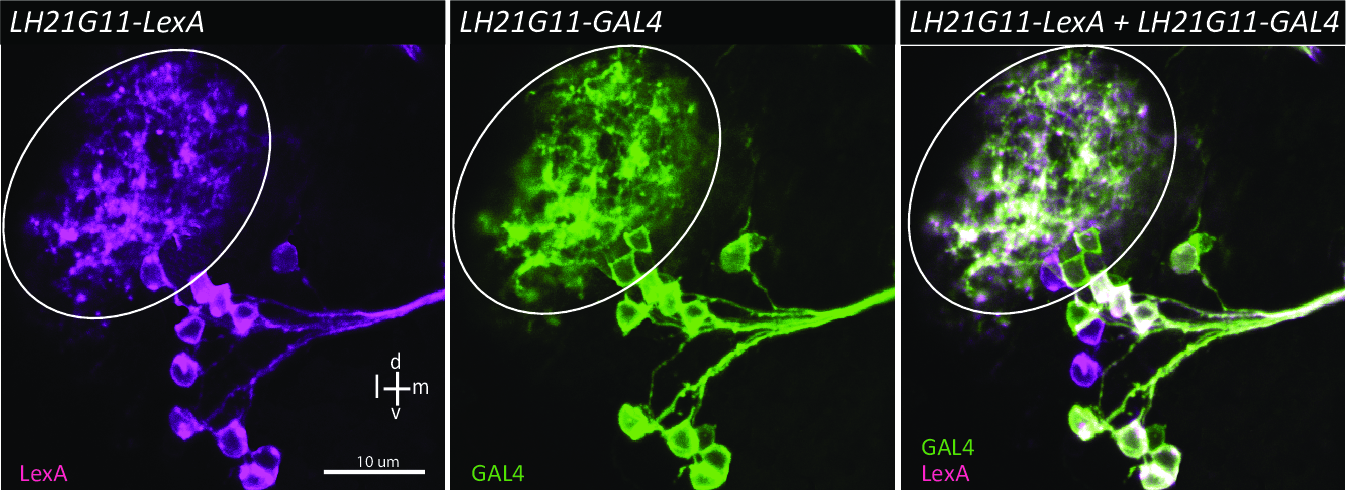

Supplement: S3 Fig — LH cells of 21G11-LexA (red) and 21G11-GAL4 (green) and the merge of both. Scale bar = 10 μm. LH, lateral horn. (TIF) [file pbio.2006749.s003.tif]

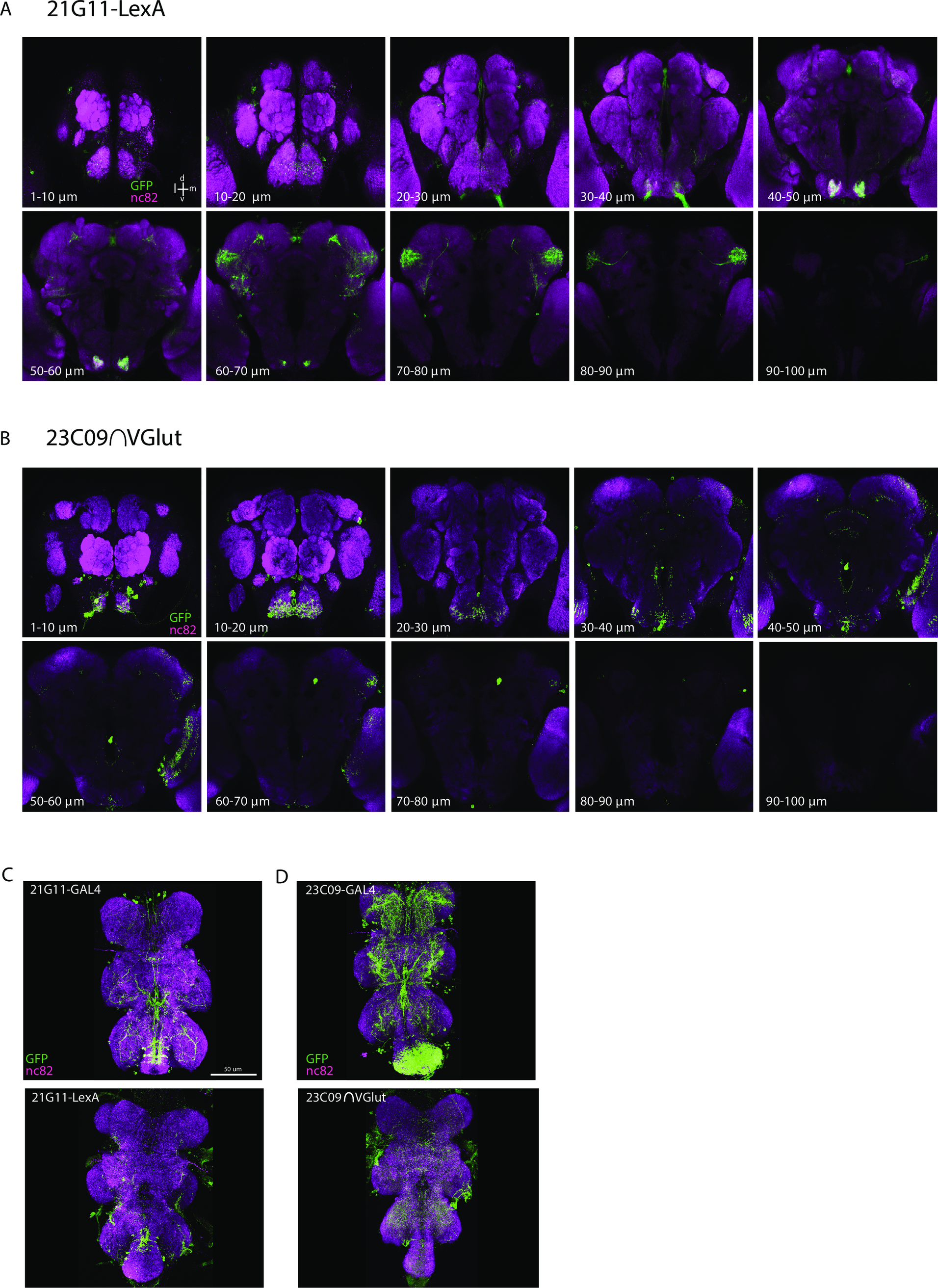

Supplement: S4 Fig — (A and B) Brain expression every 10 μm of 21G11-LexA (A) and 23C09∩VGlut (B) lines. (C) VNC expression of 21G11 and 21G11-LexA. (D) VNC expression of 23C09 and 23C09∩VGlut. For all images, green = GFP, magenta = nc82. GFP, green fluorescent protein; nc82, monoclonal antibody to Bruchpilot; VNC, ventral nerve cord. (TIF) [file pbio.2006749.s004.tif]

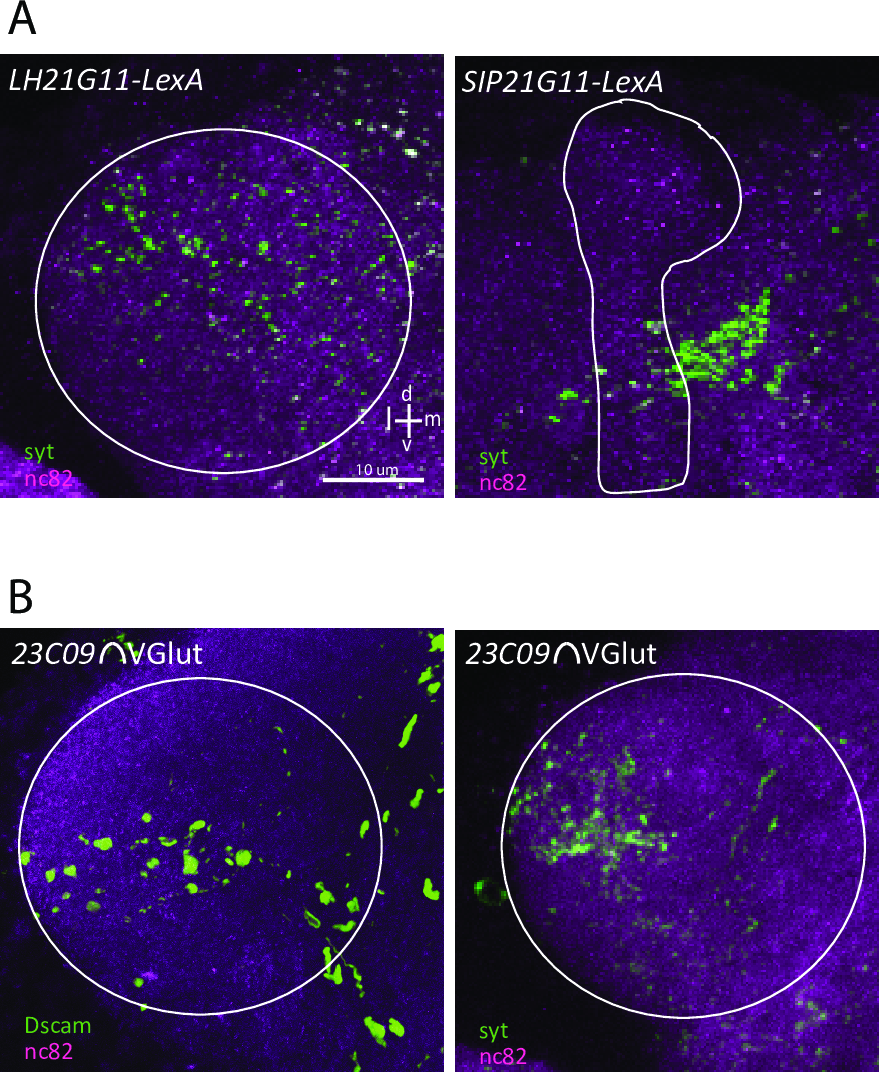

Supplement: S5 Fig — (A) Syt-HA expression in the LH and SIP of 21G11-LexA (green). The circle highlights the LH. The vertical lobe of the MB is drawn to facilitate visualization of the adjacent SIP. (B) Dscam17.1-GFP and syt-HA expression in the LH of 23C09∩VGlut (green). The circle highlights the LH. Scale bar = 10 μm. d, dorsal; Dscam17.1, Down syndrome cell adhesion molecule with isoform 1 of the transmembrane domain; GFP, green fluorescent protein; HA, hemagglutinin; l, lateral; LH, lateral horn; m, medial; MB, mushroom body; SIP, superior intermediate protocerebrum; syt, synaptotagmin; v, ventral. (TIF) [file pbio.2006749.s005.tif]

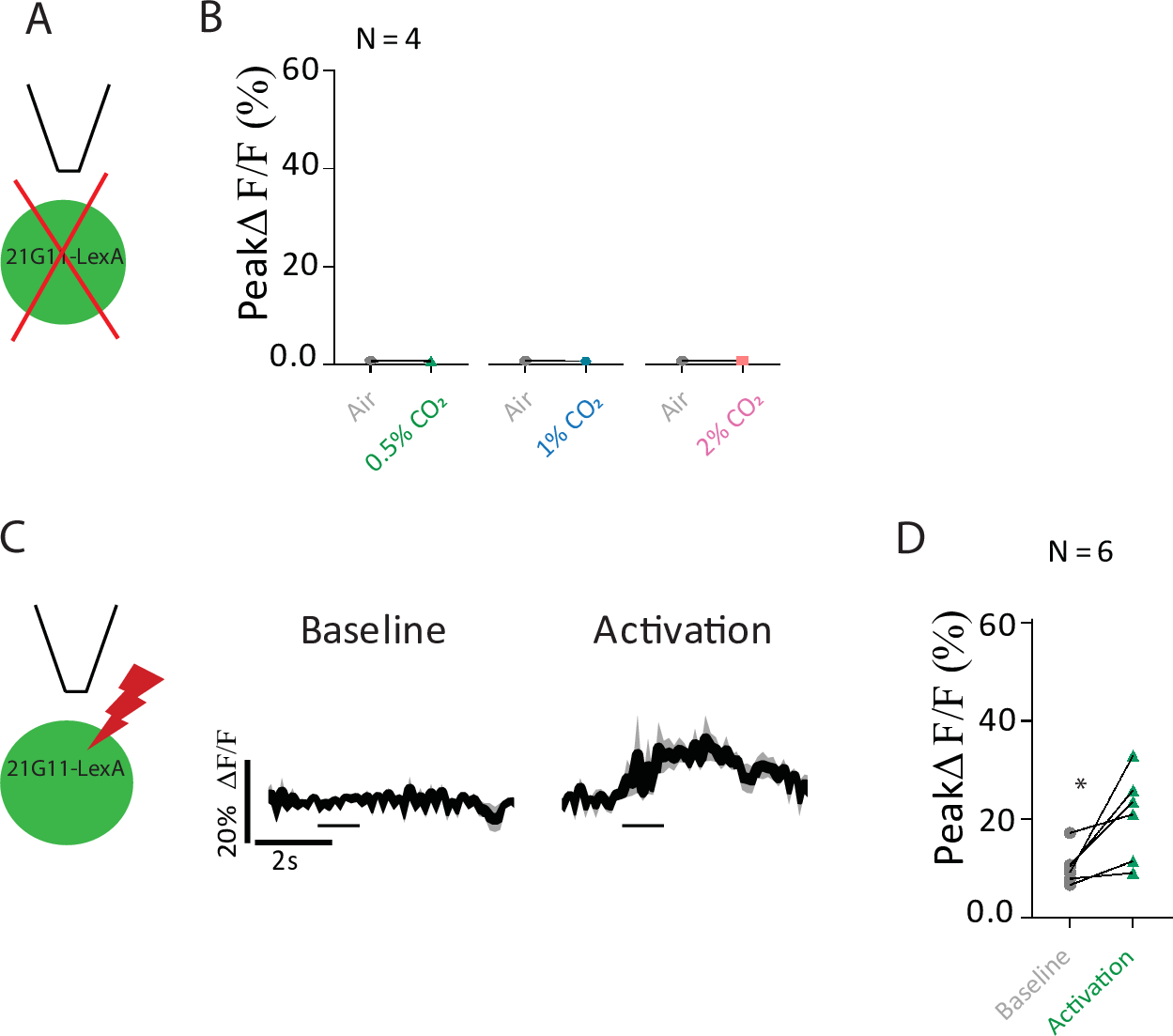

Supplement: S6 Fig — (A and B) Schematics of the experiment and calcium response at the LH, using GCaMP6m, of 21G11-LexA neurons to air, 0.5%, 1%, and 2% of CO2 while 21G11-LexA neurons are silenced by expression of Kir2.1. (C) Schematics of the experiment and LH activity of 21G11-LexA upon activation of 21G11-LexA neurons, expressing Chrimson, with 720 nm light. (D) Peak GCaMP6m intensity change upon activation. For (C), the average time course of GCaMP6m intensity change is shown. The black bar indicates the time of the stimulus. *p < 0.05. All p values are calculated with Wilcoxon signed-rank test. LH, lateral horn. (TIF) [file pbio.2006749.s006.tif]

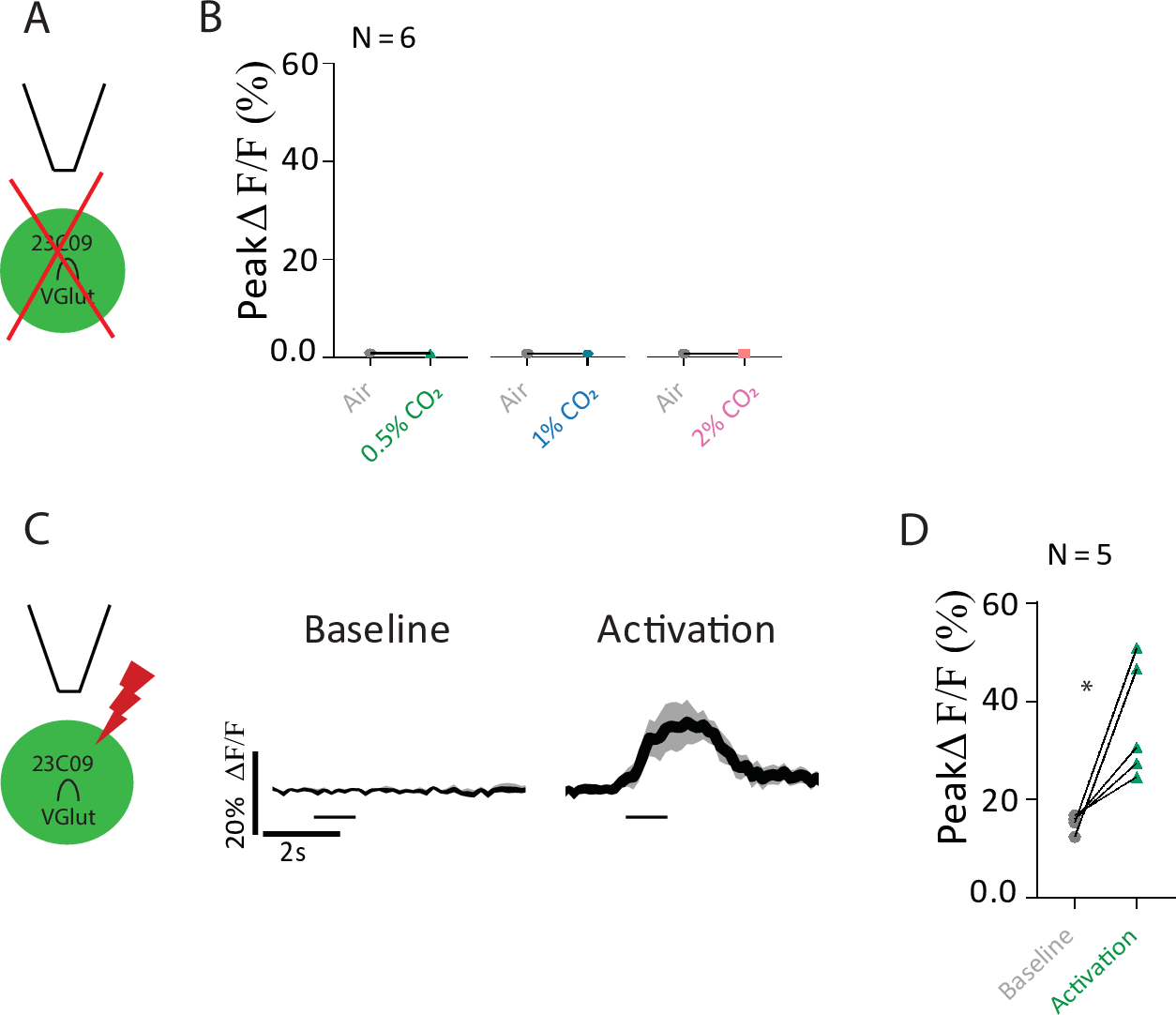

Supplement: S7 Fig — (A and B) Schematics of the experiment and calcium response at the LH, using GCaMP6m, of 23C09∩VGlut neurons to air, 0.5%, 1% and 2% of CO2, while 23C09∩VGlut neurons are silenced by expression of Kir2.1. (C) Schematics of the experiment and LH activity of 23C09∩VGlut upon activation of 23C09∩VGlut neurons, expressing Chrimson, with 720 nm light. (D) Peak GCaMP6m intensity change upon activation. For (C), the average time course of GCaMP6m intensity change is shown. The black bar indicates the time of the stimulus. *p < 0.05. All p values are calculated with Wilcoxon signed-rank test. LH, lateral horn. (TIF) [file pbio.2006749.s007.tif]

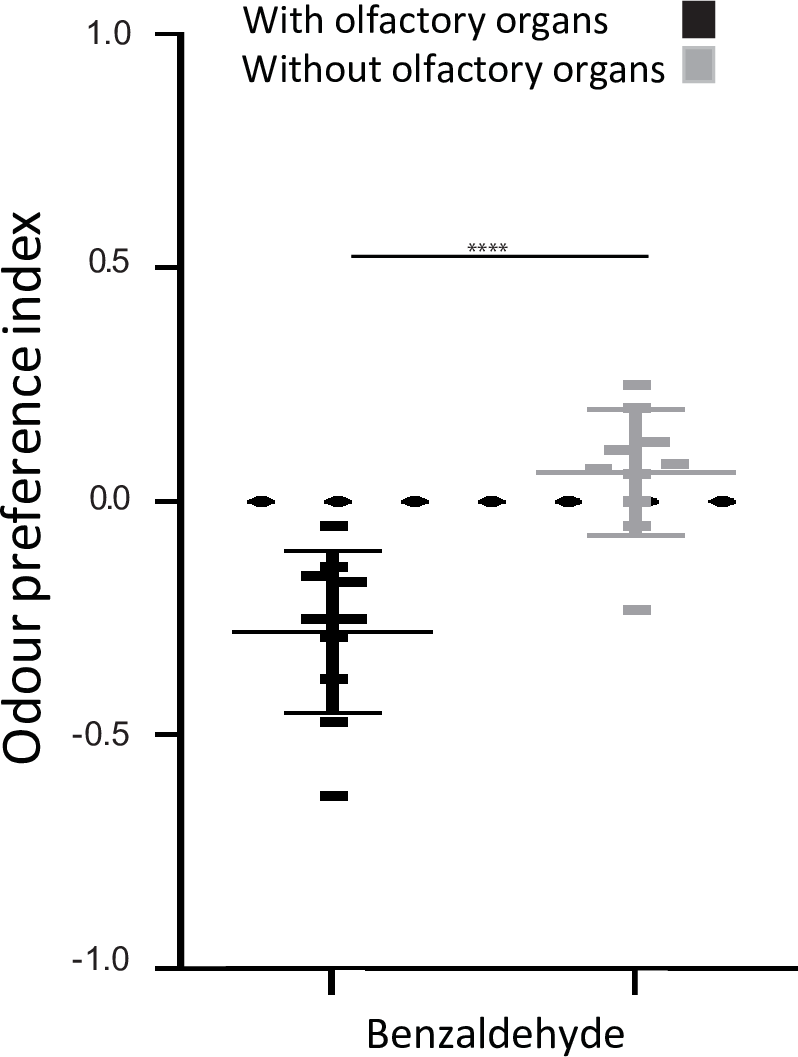

Supplement: S8 Fig — T-maze response to benzaldehyde at 1:1000 dilution (BZ). White box, flies with olfactory organs. Gray box, flies without olfactory organs. The box represents the first and the third quartiles, and the whiskers the 10th and 90th percentiles. The line across the box is the median. N = 10. Error bars indicate ±SEM ****p<0.0001. p values are calculated with Wilcoxon signed-rank test. BZ, benzaldehyde; SEM, standard error of the mean. (TIF) [file pbio.2006749.s008.tif]
